# Periodic Constrained Nuclear-Electronic Orbital Density Functional Theory for Nuclear Quantum Effects: Method Development and Application to Hydrogen Adsorption on Pt(111)

**DOI:** 10.1021/acs.jctc.5c00837

**Published:** 2025-08-11

**Authors:** Zehua Chen, Yang Yang

**Affiliations:** Theoretical Chemistry Institute and Department of Chemistry, 5228 University of Wisconsin-Madison, 1101 University Avenue, Madison, Wisconsin 53706, United States

## Abstract

We develop constrained nuclear-electronic orbital density functional theory (CNEO–DFT) with periodic boundary conditions, enabling simultaneous quantum mechanical treatment of both electrons and nuclei in extended systems at computational costs comparable to conventional DFT. Our approach employs the Gaussian-augmented plane wave framework of CP2K for both electrons and nuclei. The quantum nuclei are treated as localized, distinguishable particles, while the collective nuclear distribution satisfies periodicity. When applied to hydrogen adsorption on Pt(111), our method predicts a shift in the preferred binding site from atop (conventional DFT) to fcc hollow (CNEO–DFT), primarily due to zero-point effects. Furthermore, by capturing subtle shallow tunneling effects that enhance hydrogen mobility, CNEO–DFT shows excellent agreement with fully quantum reference calculations for differential entropy across catalytically relevant temperatures (500–800 K). The implementation also includes analytic gradients, enabling geometry optimization and molecular dynamics. This development of periodic CNEO–DFT offers an accurate and efficient framework for treating nuclear quantum effects in surfaces, interfaces, and bulk materials where hydrogen chemistry plays a crucial role.

## Graphical abstract

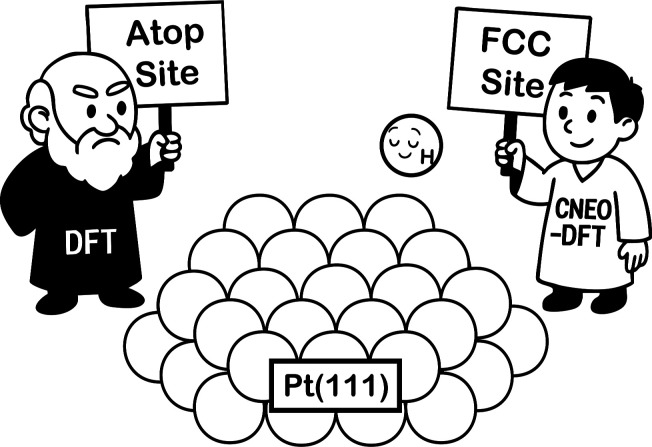

## 1 Introduction

Nuclear quantum effects (NQEs), which often manifest as zero-point energy (ZPE), nuclear delocalization, and tunneling, play a critical role in many chemical systems, particularly those involving hydrogen.^1,2^ Because of its small nuclear mass, hydrogen exhibits large ZPEs (∼0.2 eV for O–H stretching) and significant nuclear delocalization, which can substantially alter equilibrium geometries, reaction barriers, and thermodynamic properties. For example, in bulk water, quantum delocalization of protons influences the hydrogen bond network and its structural organization.^3–5^ In surface chemistry, ZPE significantly influences hydrogen adsorption and mobility on metal surfaces: it can shift preferred adsorption sites, change diffusion barriers, and alter reaction mechanisms and rates.^6–8^

The computational description of NQEs presents both theoretical and practical challenges. Exact quantum mechanical treatment requires solving the high-dimensional Schrödinger equation, which is computationally prohibitive for realistic chemical systems. For equilibrium properties, path integral molecular dynamics (PIMD)^9,10^ provides a formally exact mapping of quantum statistical mechanics onto a classical ring polymer problem. Although theoretically rigorous, PIMD typically increases computational cost by 10–50 times compared to classical molecular dynamics, limiting its application to relatively small systems or necessitating shorter simulation times. To alleviate the computational burden, various approaches have been developed, including ring-polymer contraction,^11^ multiple time-stepping,^12^ and improved thermostats.^13^

For dynamical properties, approaches like ring polymer molecular dynamics (RPMD),^14^ centroid molecular dynamics (CMD),^15,16^ and their more recent variants (thermostatted RPMD,^17,18^ quasicentroid MD^19,20^ ) extend the PIMD framework. While these methods have proven valuable, they remain computationally demanding due to the need for multiple beads per quantum particle, despite the development of acceleration techniques.^21^ The multiconfiguration time-dependent Hartree (MCTDH) method^22^ offers an alternative approach for quantum dynamics through expansion of the wave function in time-dependent basis functions. With the multilayer extensions,^23^ MCTDH is able to accurately simulate systems with tens of degrees of freedom, though it requires potential energy surfaces in specific forms and faces steep computational scaling with system size. Another highly accurate method is the quasi-adiabatic path integral (QuAPI) approach, which offers a rigorous treatment of real-time quantum dynamics in open systems with strong system-bath coupling. QuAPI explicitly tracks system-bath correlations and takes into account memory effects, making it highly accurate but computationally prohibitive for realistic chemical systems.^24,25^ Additionally, the linearized semiclassical initial value representation (LSC-IVR)^26,27^ offers another trajectory-based approach to simulate quantum dynamics and is exact in classical, high-temperature, and harmonic limits, but suffers from ZPE leakage, especially in anharmonic systems.^28–30^

For practical applications involving chemical reactions in extended systems, ZPE corrections to classical potential energy surfaces are often incorporated through ensemble-averaged variational transition state theory (EA-VTST)^31,32^ calculations. These methods provide a much more computationally efficient way to incorporate NQEs, though they rely on the accurate identification of reaction coordinates.

Recently, multicomponent quantum chemistry^33–36^ has emerged as a powerful approach for incorporating NQEs into quantum chemistry calculations and dynamical simulations. This approach enables the simultaneous quantum mechanical treatment of both electrons and nuclei. Within this framework, both multicomponent wave function methods and multicomponent density functional theory have been developed—most notably nuclear-electronic orbital coupled-cluster theory (NEO–CC)^37,38^ and nuclear-electronic orbital density functional theory (NEO–DFT).^39,40^ In NEO–DFT, NQEs and electron-quantum nuclear vibronic effects are incorporated via the self-consistent solution of coupled nuclear-electronic Kohn–Sham equations. This method has been successfully applied to a wide range of chemically relevant systems.^41–43^ Notably, a periodic implementation of NEO–DFT has been developed within the FHI-aims software package for applications to extended systems.^44^

However, in conventional NEO–DFT, nuclear wave functions are allowed to delocalize freely in search of the configuration that minimizes the multicomponent total energy. This treatment effectively eliminates the degrees of freedom associated with the quantum nuclei and leads to vibronic energy surfaces that depend only on the positions of the classical nuclei. As a result, dynamics on these vibronic surfaces become strongly nonadiabatic,^42,43^ making accurate simulations computationally demanding.

Recently, our group developed the constrained nuclear-electronic orbital density functional theory (CNEO–DFT),^45,46^ which introduces position constraints on quantum nuclear densities. These constraints enforce that the expectation values of the quantum nuclear positions match their corresponding classical coordinates, thereby preserving well-defined molecular geometries while capturing quantum delocalization effects. Unlike the conventional NEO vibronic energy surfaces, CNEO energy surfaces are full-dimensional and depend on both the positions of the classical nuclei and the constrained expectation values of the quantum nuclear positions. As a result, CNEO overcomes the high nonadiabaticity challenges inherent to the conventional NEO framework. The full-dimensional CNEO surfaces resemble conventional Born–Oppenheimer potential energy surfaces, but inherently include quantum nuclear delocalization and ZPE contributions.

Within the CNEO framework, both CNEO–DFT and CNEO molecular dynamics (CNEO-MD)^47,48^ have demonstrated significant improvements over conventional DFT-based methods for both static and dynamical properties across a wide range of chemical systems. These properties include equilibrium geometries,^49,50^ vibrational frequencies,^49,51,52^ reaction rate constants,^53,54^ and nonadiabatic dynamics properties.^55^ Notably, both CNEO–DFT and CNEO-MD retain computational efficiency comparable to conventional DFT and DFT-based *ab initio* molecular dynamics,^49,52^ making the CNEO framework an accurate and practical approach for incorporating NQEs into electronic structure calculations and dynamical simulations.

However, previous implementations of CNEO–DFT have been limited to molecular systems and are not directly applicable to extended systems such as surfaces, interfaces, and bulk materials, which require a periodic treatment. To incorporate NQEs in such systems, researchers have typically relied on conventional periodic DFT calculations with *a posteriori* ZPE corrections,^56–58^ or alternatively, on path-integral simulations facilitated by machine learning potentials trained on DFT data.^59,60^

In this work, we present a development of CNEO–DFT with periodic boundary conditions (PBC) within the Gaussian-augmented plane wave (GAPW) framework^61,62^ of the CP2K software package.^63^ This development enables the efficient quantum mechanical treatment of both electrons and nuclei in periodic systems, with a computational cost comparable to that of conventional DFT calculations. Additionally, our implementation includes analytic gradients with respect to both classical nuclear coordinates and quantum nuclear expectation positions, enabling efficient geometry optimizations, transition state search, and molecular dynamics simulations.

To demonstrate the capabilities and advantages of periodic CNEO–DFT, we apply it to the prototypical problem of hydrogen adsorption on the Pt(111) surface—a system of fundamental importance in heterogeneous catalysis. Our results show that NQEs significantly influence the preferred adsorption sites: conventional DFT incorrectly favors the atop site, whereas methods incorporating ZPE—either via *a posteriori* correction to conventional DFT or directly through CNEO–DFT—correctly identify the fcc hollow site as most stable. Moreover, CNEO–DFT yields significantly improved estimates of the differential entropy associated with hydrogen adsorption, exhibiting excellent agreement with fully quantum reference calculations, particularly within the temperature range relevant to catalytic applications.

The remainder of this paper is organized as follows. Section 2 presents the theoretical development of periodic CNEO–DFT within the GAPW formalism. Section 3 presents the computational details for studying H/Pt(111). Section 4 discusses the results and demonstrates the impact of NQEs on adsorption site preferences and thermodynamic properties. Finally, Section 5 concludes with a summary and outlook for future applications.

## 2 Theory

In this section, we will begin with reviewing the CNEO–DFT theory, with an emphasis on fundamental assumptions, energy functional, and coupled Kohn–Sham equations. Then we will continue with the adaptation of CNEO–DFT to periodic systems through the GAPW method, and end with the self-consistent field procedure and analytic gradients needed for structural optimization and molecular dynamics simulations.

### 2.1 Constrained Nuclear-Electronic Orbital Density Functional Theory

CNEO belongs to the multicomponent quantum theory,^33–36^ which provides a rigorous framework for simultaneous quantum mechanical treatment of both electrons and nuclei. Within this framework, NEO–DFT^36^ has emerged as a powerful approach for modeling molecular systems and has been extended for periodic systems within the FHI-aims software package.^44^ As noted earlier, while conventional NEO–DFT captures electron-quantum nuclear vibronic effects, it loses the degree of freedom for the quantum nuclei. This results in vibronic energy surfaces that depend only on the classical nuclear positions, and necessitates strongly nonadiabatic dynamics that are computationally demanding. CNEO–DFT addresses this challenge by applying position constraints on quantum nuclei and anchoring their expectation values to classical coordinates while preserving their quantum character.

A key treatment that enables practical calculations of CNEO–DFT is the distinguishable-particle approximation. This approximation is physically motivated by the fact that in almost all systems of chemical interest, quantum nuclear wave functions are highly localized with minimal overlap between neighboring nuclei. As such, each quantum nucleus can be treated with its own single-particle density

$$\rho_{a}^{n}\left(r\right)={\left|\psi_{g.s.}^{n,a}\left(r\right)\right|}^{2}$$ 1

Here ρ_*a*_^n^ denotes the single-particle density of the *a*th quantum nucleus, and ψ_g.s._^n,*a*^ is the associated normalized ground-state nuclear orbital.

The position constraint, which ensures the expectation position of the *a*th quantum nucleus to match its classical coordinate ***R***_*a*_, is

$$\left\langle {\mathrm{r̂}}_{a}^{n}\right\rangle \equiv \int dr\rho_{a}^{n}\left(r\right)r=R_{a}$$ 2

Through this constraint mechanism, CNEO is able to maintain well-defined molecular structures essential for chemical interpretation. By varying the constrained nuclear expectation positions and solving for electron and nuclear orbitals that minimize the total energy, one can construct effective energy surfaces that inherently include NQEs, particularly quantum nuclear delocalization.^47^ We have shown in the past that these energy surfaces can be used in classical molecular dynamics simulations to accurately predict static and dynamic properties associated with light nuclei, especially hydrogen, for which NQEs are significant.^47,48^

For a system with both classical and quantum nuclei, the CNEO–DFT energy functional can be written as^45^

$$\begin{array}{ll} E_{V_{ext}}\left[\rho^{e},\left\{\rho_{a}^{n}\right\}\right] & =T_{s}^{e}\left[\rho^{e}\right]+\underset{a}{\sum }T_{s}^{n,a}\left[\rho_{a}^{n}\right]\\ & +E_{ext}^{e}\left[\rho^{e}\right]+\underset{a}{\sum }E_{ext}^{n,a}\left[\rho_{a}^{n}\right]\\ & +E_{H}\left[\rho^{e},\left\{\rho_{a}^{n}\right\}\right]+E_{xc}^{e}\left[\rho^{e}\right]+E_{c}\left[\rho^{e},\left\{\rho_{a}^{n}\right\}\right] \end{array}$$ 3

where *T*_s_^e^ and *T*_s_^n,*a*^ are the noninteracting kinetic energies for electrons and the *a*th quantum nucleus, respectively. *E*_ext_ represents the interaction with the external potential, typically the Coulombic potential from classical nuclei

4 $$E_{ext}^{e}\left[\rho^{e}\right]=\int dr\rho^{e}\left(r\right)V_{ext}\left(r\right)$$

$$E_{ext}^{n,a}\left[\rho_{a}^{n}\right]=-Z_{a}\int dr\rho_{a}^{n}\left(r\right)V_{ext}\left(r\right)$$ 5

where *Z* *_a_* is the charge of the *a*th quantum nucleus. *E*_H_ is the Hartree energy, *E*_xc_^e^ is the electronic exchange–correlation energy, and *E*_c_ is the multicomponent correlation energy.

The Hartree energy, with nuclear self-interaction terms excluded, is expressed as

$$\begin{array}{ll} E_{H}\left[\rho^{e},\left\{\rho_{a}^{n}\right\}\right] & =\frac{1}{2}\int dr\int dr^{\prime }\frac{\rho^{e}\left(r\right)\rho^{e}\left(r^{\prime }\right)}{\left|r-r^{\prime }\right|}\\ & -\underset{a}{\sum }Z_{a}\int dr\int dr^{\prime }\frac{\rho^{e}\left(r\right)\rho_{a}^{n}\left(r^{\prime }\right)}{\left|r-r^{\prime }\right|}\\ & +\frac{1}{2}\underset{a\neq b}{\sum }Z_{a}Z_{b}\int dr\int dr^{\prime }\frac{\rho_{a}^{n}\left(r\right)\rho_{b}^{n}\left(r^{\prime }\right)}{\left|r-r^{\prime }\right|} \end{array}$$ 6

The multicomponent correlation energy can be approximated using a two-body expansion that considers only pairwise correlations between electrons and each quantum nucleus, employing existing electron–proton correlation (epc) functionals.^39,40,64^ This approximation takes the form

$$E_{c}\left[\rho^{e},\left\{\rho_{a}^{n}\right\}\right]\approx \underset{a}{\sum }E_{epc}\left[\rho^{e},\rho_{a}^{n}\right]$$ 7

However, in this work, we will neglect this multicomponent correlation term, as previous tests on vibrational spectroscopy^85^ and reaction rate constants^53^ have shown that existing epc functionals have only a minor impact on the results. The implementation and investigation of multicomponent correlation for periodic systems are left for future studies.

By minimizing the energy functional subject to the orbital normalization and nuclear expectation value constraints, the coupled Kohn–Sham equations for electrons and quantum nuclei can be derived^45^

8 $$\left[-\frac{1}{2}\nabla^{2}+V_{eff}^{e}\left(r\right)\right]\psi_{i}^{e}\left(r\right)=\varepsilon_{i}^{e}\psi_{i}^{e}\left(r\right)$$

$$\left[-\frac{1}{2M_{a}}\nabla^{2}+V_{eff}^{n,a}\left(r\right)+f_{a}\cdot \left(r-R_{a}\right)\right]\psi_{i}^{n,a}\left(r\right)=\varepsilon_{i}^{n,a}\psi_{i}^{n,a}\left(r\right)$$ 9

where the effective potentials *V*_eff_^e^ and *V*_eff_^n,*a*^ include the external potential, Hartree potentials, and (exchange-)­correlation potentials. In the nuclear Kohn–Sham equation, the self-interaction contribution within the nuclear effective potential is explicitly removed. Additionally, the nuclear equation includes a term ***f***_*a*_·(***r*** – ***R***_*a*_), which arises from the Lagrange multiplier enforcing the position constraint. The multiplier ***f***_*a*_ must be iteratively optimized to ensure $\left\langle {\mathrm{r̂}}_{a}^{n}\right\rangle =R_{a}$ for each quantum nucleus.

### 2.2 Extension to Periodic Boundary Conditions

Extending CNEO–DFT to periodic systems raises a conceptual question: how can the distinguishable-particle approximation be reconciled with lattice periodicity? In conventional electronic structure theory with PBC, electronic wave functions form Bloch states, which are nonsquare-integrable and delocalized across the entire crystal. In contrast, within the CNEO framework, each quantum nucleus is represented by a localized square-integrable wave function with a well-defined position expectation value. This localized treatment applies not only to quantum nuclei of the same type within a unit cell but also to those related by lattice translations, thus raising a valid concern regarding a potential violation of periodicity.

The resolution to this concern lies in recognizing that the CNEO framework does not enforce periodicity at the level of individual quantum nuclear densities, but rather through their collective distribution. Specifically, the total nuclear density for nuclear species “n”, defined as

$$\rho^{n}\left(r\right)=\underset{a\in unitcell}{\sum }\;\underset{n}{\sum }\rho_{a}^{n}\left(r+T_{n}\right)$$ 10

satisfies the periodicity

$$\rho^{n}\left(r+T_{n}\right)=\rho^{n}\left(r\right)$$ 11

where ***n*** = (*n*_1_, *n*_2_, *n*_3_) and ***T***_***n***_ = *n*_1_ ***a***_1_ + *n*_2_ ***a***_2_ + *n*_3_ ***a***_3_ represents a lattice vector. Note that here each ρ_*a*_^n^ is localized around its corresponding nuclear expectation position, while the total density ρ^n^ spans the entire space and satisfies periodicity. In practice, since quantum nuclei in most chemically relevant systems exhibit negligible spatial overlap, the distinguishable-particle approximation is highly accurate. Under this treatment, the nuclear Kohn–Sham equations only need to be solved for quantum nuclei within a single unit cell. This formulation offers significant computational advantages: it eliminates the need to construct nuclear Bloch states while still preserving the translational symmetry required for extended systems.

We further note that the distinguishable-particle approach aligns well with existing simplifications in the previous periodic NEO–DFT implementation.^44^ In that work, the indistinguishable-particle implementation in FHI-aims employs a Γ-point approximation for nuclear *k*-points sampling and uses local exchange to cancel nuclear self-interactions, thereby avoiding the need to calculate the computationally intensive global exchange required for delocalized nuclear wave functions. These approximations, in essence, reflect a distinguishable-particle treatment that takes advantage of the highly localized nature of quantum nuclei. In contrast, our explicit distinguishable-particle formalism here provides a more straightforward pathway to these simplifications.

### 2.3 Electrostatic Energy Evaluation

For periodic systems, the evaluation of Coulomb interactions poses a significant computational challenge due to their inherently long-range nature. The total electrostatic energy per unit cell, denoted *E*_elst_, can be expressed as

$$\begin{array}{ll} E_{elst} & =E_{H}\left[\rho^{e},\left\{\rho_{a}^{n}\right\}\right]+E_{ext}^{e}\left[\rho^{e}\right]\\ & +\underset{a\in unitcell}{\sum }E_{ext}^{n,a}\left[\rho_{a}^{n}\right]+E_{nuc} \end{array}$$ 12

where *E*_H_[ρ^e^, {ρ_*a*_^n^}], defined slightly differently from eq 6 for molecular systems, represents the Hartree energy per unit cell for the periodic system. The terms *E*_ext_^e^[ρ^e^] and *E*_ext_^n,*a*^[ρ_*a*_^n^] are the external potential energies per unit cell due to classical nuclei acting on the electronic and quantum nuclear densities, respectively. The final term *E*_nuc_ accounts for the classical nuclear repulsion energy per unit cell.

The Hartree contributions from the quantum nuclear and electronic densities will be addressed in the next section. Here, we focus on efficiently evaluating the terms involving classical nuclear potentials using the Ewald summation technique.^65^

The Ewald method separates the Coulomb interactions into short-range and long-range components and evaluates them in real and reciprocal space, respectively. This is achieved by representing each classical point charge as a Gaussian-smeared charge distribution

$$\rho^{A}\left(r\right)=-\frac{Z_{A}}{{\left(R_{A}^{c}\right)}^{3}}\pi^{-3/2}\;\exp\left[-{\left(\frac{r-R_{A}}{R_{A}^{c}}\right)}^{2}\right]$$ 13

where *Z*_*A*_ is the charge of classical nucleus *A*, ***R***_*A*_ is its position, and *R*_*A*_^c^ is the smearing width. These Gaussian densities reproduce the same long-range electrostatic potential as point charges while enabling more efficient evaluation of Coulombic energies. Combining these Gaussian-smeared classical nuclear densities with the electronic and quantum nuclear contributions leads to a total charge density defined as

$$\rho_{tot}=\rho^{e}\left(r\right)-\underset{a\in unitcell}{\sum }Z_{a}\underset{n}{\sum }\rho_{a}^{n}\left(r+T_{n}\right)+\underset{A}{\sum }\rho^{A}\left(r\right)$$ 14

where the second term accounts for the periodic replication of the quantum nuclear densities as discussed earlier, and the third term sums over all smeared classical nuclei in the periodic system. The Hartree energy per unit cell associated with this total charge density, *E*_H_[ρ_tot_], can be evaluated efficiently using the GAPW approach detailed in the next section. However, before introducing the GAPW approach, we note that because the short-range behavior of the Gaussian-smeared ions differs from that of true point charges, correction terms must be included to account for these discrepancies. After regrouping the terms, the total electrostatic energy per unit cell can be rewritten as

15 $$\begin{array}{ll} E_{elst} & =E_{H}\left[\rho_{tot}\right]-\sum_{a\in unitcell}E_{H}\left[Z_{a}\rho_{a}^{n}\right]+E_{short}^{e}\left[\rho^{e}\right]\\ & +\sum_{a\in unitcell}E_{short}^{n,a}\left[\rho_{a}^{n}\right]-E_{self}+E_{ovrl} \end{array}$$

Here the term −*E*_H_[*Z*_*a*_ρ_*a*_^n^] removes quantum nuclear self-interactions. *E*_short_^e^[ρ^e^] and *E*_short_^n,*a*^[ρ_*a*_^n^] correct for discrepancies between Gaussian and point-charge interactions, and they are evaluated as

16 $$E_{short}^{e}\left[\rho^{e}\right]=\int dr\rho^{e}\left(r\right)V_{ext}^{short}\left(r\right)$$

$$E_{short}^{n,a}\left[\rho_{a}^{n}\right]=-Z_{a}\int dr\rho_{a}^{n}\left(r\right)V_{ext}^{short}\left(r\right)$$ 17

where the short-range external potential is given by

$$V_{ext}^{short}\left(r\right)=-\underset{A}{\sum }\frac{Z_{A}}{\left|r-R_{A}\right|}erfc\left(\frac{\left|r-R_{A}\right|}{R_{A}^{c}}\right)$$ 18

The complementary error function erfc captures the discrepancy between the short-range behavior of Gaussian-smeared and point-charge Coulomb potentials. Its exponential decay ensures that the resulting short-range integrals can be efficiently evaluated in real space using a modest cutoff radius. The terms *E*_self_ and *E*_ovrl_ are correction terms that arise naturally from the Ewald summation technique (details in the Supporting Information; see also refs 63 and 65 ).

All terms in the reformulated electrostatic energy expression—except for *E*_H_[ρ_tot_]—can be computed efficiently using established methods. It is worth noting that although the removal of quantum nuclear self-interactions, −*E*_H_[*Z*_*a*_ρ_*a*_^n^], is formally included in the expression, this term does not need to be evaluated explicitly in practice, as will be discussed in the following section.

### 2.4 Gaussian-Augmented Plane Wave Method with Quantum Nuclei

We now turn to the GAPW method, which provides an efficient framework for evaluating the *E*_H_[ρ_tot_] term introduced in the previous section. The GAPW method was originally developed to address the dual character of electronic charge densities: they are smooth in the interstitial regions but exhibit sharp peaks near atomic nuclei. This contrasting behavior makes methods relying solely on either real-space or reciprocal-space representations inefficient.

The GAPW method^61,62^ addresses this challenge through a spatial partitioning scheme that divides the system into atomic regions *U*_*A*_ (spherical regions centered on each atom) and an interstitial region *I*. This partitioning enables computational treatments tailored to the characteristics of each region: local numerical integration is used within atomic regions, where the charge density varies rapidly, while fast Fourier transforms (FFT) are employed in the interstitial region, where the density is smooth and slowly varying.

The electronic density is decomposed as

19 $$\rho^{e}\left(r\right)={\mathrm{\rho ̃}}^{e}\left(r\right)+\sum_{A}\rho_{A}^{e,1}\left(r\right)-{\mathrm{\rho ̃}}_{A}^{e,1}\left(r\right)$$

where ${\mathrm{\rho ̃}}^{e}$ is the smooth global electronic density, ρ_*A*_^e,1^ denotes the sharply peaked local (or “hard”) density around atom *A*, and ${\mathrm{\rho ̃}}_{A}^{e,1}$ is its corresponding smooth (or “soft”) approximation.

The decomposition is constructed to satisfy the following spatial requirements:

- within each atomic region *U*_*A*_: ρ_*A*_^e,1^(***r***) = ρ^e^(***r***) for ***r*** ∈ *U*_*A*_;
- in the interstitial region *I*: ${\mathrm{\rho ̃}}^{e}\left(r\right)=\rho^{e}\left(r\right)$ for ***r*** ∈ *I*;
- the local soft density ${\mathrm{\rho ̃}}_{A}^{e,1}$ acts as a bridging function: ${\mathrm{\rho ̃}}_{A}^{e,1}\left(r\right)=\rho_{A}^{e,1}\left(r\right)$ for ***r*** ∉ *U*_*A*_ and ${\mathrm{\rho ̃}}_{A}^{e,1}\left(r\right)={\mathrm{\rho ̃}}^{e}\left(r\right)$ for ***r*** ∈ *U*_*A*_.

For quantum nuclei, a similar decomposition is applied to facilitate efficient numerical treatment

$$\rho_{a}^{n}\left(r\right)={\mathrm{\rho ̃}}_{a}^{n}\left(r\right)+\left[\rho_{a}^{n}\left(r\right)-{\mathrm{\rho ̃}}_{a}^{n}\left(r\right)\right]$$ 20

where ${\mathrm{\rho ̃}}_{a}^{n}$ represents the smooth tail that extends into the interstitial region, and $\rho_{a}^{n}-{\mathrm{\rho ̃}}_{a}^{n}$ captures the sharply varying core component localized within the atomic region.

In practice, the decomposition is implemented by classifying nuclear Gaussian basis functions based on their exponents, angular momenta, and the radius of the atomic region. Specifically, only primitive Gaussian functions whose values at the boundary of the atomic sphere exceed a specified threshold are designated as “soft” and contribute to the smooth tail density ${\mathrm{\rho ̃}}_{a}^{n}$ . For regular nuclei, including protons—the lightest stable nuclei—the nuclear densities are typically highly localized, and accordingly, localized nuclear basis functions are commonly used. As a result, only a small number of basis functions contribute to the soft component. As a reference, for the quantum protons studied in this work, we found that the soft components contribute less than 0.01% of the total nuclear density and can therefore be safely neglected. However, the soft component may become non-negligible in systems involving lighter exotic atom such as muonium.

Another key feature of the GAPW method is the introduction of compensation charge. The total compensation charge density, denoted ρ^0^, is expressed as a sum of atom-centered local compensation charge densities

21 $$\rho^{0}\left(r\right)=\sum_{A}\rho_{A}^{0}\left(r\right)$$

Each local compensation charge density ρ_*A*_^0^(***r***), centered at atom *A*, is constructed to reproduce the multipole moments of a specified target charge distribution ρ_*A*_^tgt^(***r***) in its vicinity. This is achieved in two steps. First, the multipole moments of the target charge distribution are computed as

22 $$Q_{A}^{lm}=q_{lm}\left[\rho_{A}^{tgt}\right]\equiv \frac{4\pi }{2l+1}\int_{0}^{2\pi }d\phi \int_{0}^{\pi }d\theta \;\sin\theta \int_{0}^{\infty }dr{\;r}^{l+2}\rho_{A}^{tgt}\left(r,\theta ,\phi \right)S_{l}^{m}\left(\theta ,\phi \right)$$

where *S*_*l*_^*m*^ are real spherical harmonics, and the origin of the spherical coordinate system is placed at the atomic center *A*. Second, the compensation charge ρ_*A*_^0^(***r***) is expressed as a linear combination of spherical Gaussian functions *g*_*A*_^*lm*^ using the computed multipole moments

23 $$\rho_{A}^{0}\left(r\right)=\sum_{l=0}^{\infty }\;\sum_{m=-l}^{l}Q_{A}^{lm}g_{A}^{lm}\left(r\right)$$

In the context of CNEO–DFT, classical and quantum nuclei are treated differently, which results in slightly different forms for the target charge densities. Specifically, for classical nuclei, the target charge density is given by $\rho_{A}^{tgt}=\rho_{A}^{e,1}-{\mathrm{\rho ̃}}_{A}^{e,1}+\rho^{A}$ , which accounts for the difference between the hard and soft electronic densities as well as the smeared classical nuclear charge. In contrast, for quantum nuclei, the target charge density is defined as $\rho_{a}^{tgt}=\rho_{a}^{e,1}-{\mathrm{\rho ̃}}_{a}^{e,1}-Z_{a}\left(\rho_{a}^{n}-{\mathrm{\rho ̃}}_{a}^{n}\right)$ , which captures both the electronic and nuclear density differences between hard and soft components.

With these compensation charge densities defined, the residual distribution ρ_*A*_^tgt^(***r***) – ρ_*A*_^0^(***r***) is guaranteed to have zero Coulomb interaction with any external charge distribution outside the atomic region *U*_*A*_. This property enables a clean and rigorous separation of the total Hartree energy into global and local contributions^61,62^

$$\begin{array}{ll} E_{H}\left[\rho_{tot}\right] & =E_{H}\left[{\mathrm{\rho ̃}}_{tot}\right]+\underset{\begin{array}{l} A\in classical\\ A\in unitcell \end{array}}{\sum }E_{H}\left[\rho_{A}^{e,1}+\rho^{A}\right]-E_{H}\left[{\mathrm{\rho ̃}}_{A}^{e,1}+\rho_{A}^{0}\right]\\ & +\underset{a\in unitcell}{\sum }E_{H}\left[\rho_{a}^{e,1}-Z_{a}\rho_{a}^{n}\right]-E_{H}\left[{\mathrm{\rho ̃}}_{a}^{e,1}-Z_{a}{\mathrm{\rho ̃}}_{a}^{n}+\rho_{a}^{0}\right] \end{array}$$ 24

where ${\mathrm{\rho ̃}}_{tot}$ is the soft component of the total charge density, given by

$${\mathrm{\rho ̃}}_{tot}={\mathrm{\rho ̃}}^{e}\left(r\right)-\underset{a\in unitcell}{\sum }Z_{a}\underset{n}{\sum }{\mathrm{\rho ̃}}_{a}^{n}\left(r+T_{n}\right)+\rho^{0}\left(r\right)$$ 25

The first term $E_{H}\left[{\mathrm{\rho ̃}}_{tot}\right]$ in eq 24 corresponds to the Hartree energy of the smooth global charge distribution. Although it is long-range, it can be efficiently evaluated in reciprocal space using FFT. The remaining terms involve localized charge densities and are handled via direct numerical integration on atom-centered real-space grids.

We note that in this formulation, the quantum nuclear self-interaction term does not need to be evaluated explicitly. Specifically, by using the identity

$$E_{H}\left[\rho_{a}^{e,1}-Z_{a}\rho_{a}^{n}\right]-E_{H}\left[Z_{a}\rho_{a}^{n}\right]=E_{H}\left[\rho_{a}^{e,1}\right]-Z_{a}\iint dr\;dr^{\prime }\frac{\rho_{a}^{e,1}\left(r\right)\rho_{a}^{n}\left(r^{\prime }\right)}{\left|r-r^{\prime }\right|}$$ 26

we eliminate the need to compute the nuclear self-interaction and instead evaluate only the electron–nuclear attraction term.

### 2.5 Self-Consistent Field and Analytic Gradients

The final essential components for practical CNEO–DFT calculations are an efficient self-consistent field (SCF) procedure and analytic gradients, which are required for structure optimization and molecular dynamics simulations. These capabilities enable routine inclusion of NQEs at a computational cost comparable to that of conventional DFT.

The SCF procedure centers on solving the coupled Kohn–Sham equations for both electrons and quantum nuclei. For the electronic system, we adapt the standard GAPW Hamiltonian^61,62^ to include the effects of quantum nuclear densities. This adaptation primarily involves modifying the Hartree potential and updating the compensation charges ρ^0^ to account for the quantum nuclear charge distributions. For the quantum nuclei, the Hamiltonian includes their kinetic energy terms (scaled by nuclear masses), Coulomb interactions with electrons, classical nuclei, and other quantum nuclei, as well as the position constraint terms enforced by Lagrange multipliers. The explicit matrix elements for both electronic and nuclear Hamiltonians within the GAPW framework are provided in the Supporting Information.

Our implementation integrates nuclear and electronic calculations in a simultaneous manner, as illustrated in Figure 1. The process begins with an initialization step where the electronic system is solved under the potential generated by standard Gaussian-smeared nuclear charge distributions centered at both classical and quantum nuclear positions. In subsequent iterations, the procedure alternates between nuclear and electronic calculations as follows:

1. after each electronic iteration, nuclear Kohn–Sham Hamiltonians are constructed using the updated electronic density and nuclear densities, or standard Gaussian-smeared nuclear charges if this is the first iteration;
2. for each quantum nucleus, the Lagrange multiplier ***f***_*a*_ is iteratively optimized to solve for the ground-state nuclear density that satisfies the position constraint. This step requires multiple diagonalizations of the nuclear Kohn–Sham equations. To reduce computational cost, only the Lagrange multiplier ***f***_*a*_ is updated during this step, while the rest of the nuclear Hamiltonian, which in principle depends on the updated nuclear orbitals, is kept fixed;
3. the nuclear densities are updated, and the electronic Hamiltonian is accordingly modified through changes to the global and local Hartree potentials, as well as the compensation charges;
4. a single electronic Kohn–Sham iteration is then performed. This update step can utilize the history of electronic orbitals from previous iterations, along with acceleration techniques such as direct inversion in the iterative subspace (DIIS),^66^ orbital transformation (OT),^67^ and density mixing;
5. convergence of the electronic density matrix is checked before proceeding to the next iteration.

**Figure 1:**
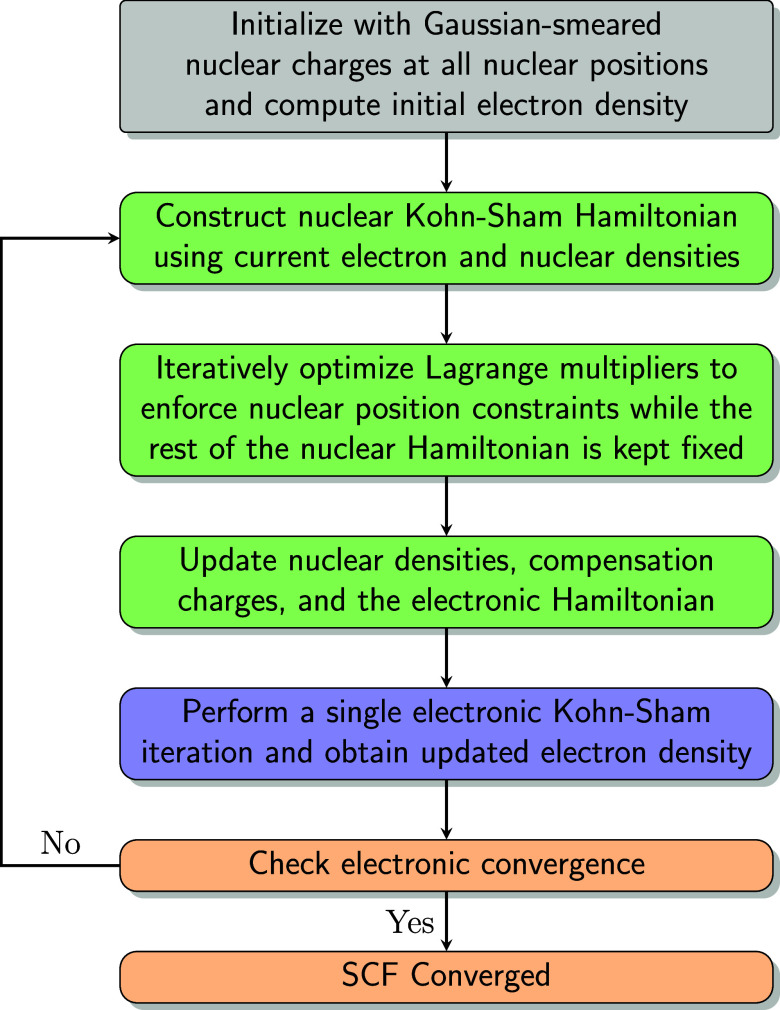
Schematic representation of the CNEO–DFT self-consistent field procedure. The process begins with electronic initialization using classical smeared ions, followed by alternating nuclear and electronic steps. The nuclear calculations include iterative optimization of the Lagrange multiplier for position constraints, while the rest of the nuclear Hamiltonian is temporarily kept fixed in this iteration. The electronic step may employ acceleration techniques such as DIIS, OT, or density mixing.

We note that while a simultaneous DIIS approach^68^ for both electrons and nuclei is more favorable in molecular systems and is implemented in our molecular code, here for periodic systems, we adopt the approach described above that only applies acceleration to the electronic system to ensure compatibility with CP2K’s exiting electronic SCF algorithms. A key aspect of our implementation is the avoidance of separate SCF cycles for the subsystems. Specifically, instead of fully converging the nuclear subsystem through repeated updates of the nuclear densities and the potentials, we perform the position constraint optimization using a nuclear Hamiltonian in which only the Lagrange multiplier ***f***_*a*_ is updated. The nuclear density is updated only after the position constraint is satisfied, just before the subsequent electronic step. This strategy maintains a high computational efficiency while preserving the simultaneous convergence of the coupled electron–nuclear system.

The convergence criterion for the overall SCF procedure is primarily based on the electronic density matrix, since with the outlined procedure, a tight convergence in the electronic subsystem naturally implies minimal changes in the nuclear subsystem. Position constraints are enforced at each iteration to ensure that the quantum nuclear expectation values remain properly satisfied.

The computational overhead of CNEO–DFT relative to conventional DFT calculations is minimal within each SCF iteration. The nuclear-specific energy terms are seamlessly integrated into the existing GAPW framework without introducing any additional computationally intensive operations. In particular, the most demanding components involving nuclear densities, such as contributions to the electrostatic energy are incorporated directly into the existing FFT-based Hartree solver and grid-based integration schemes, which are almost identical to those used in standard all-electron GAPW calculations. Moreover, the cost of solving nuclear eigenvalue problems remains minor compared to that of the electronic subsystem, owing to the use of the distinguishable-particle approximation.

In the current development, acceleration techniques such as DIIS, OT, and density mixing are only applicable to the electronic subsystem. As a result, convergence typically requires a few more SCF iterations than conventional DFT, leading to a moderate increase in overall computational cost. Future development of simultaneous orbital transformation schemes for multicomponent systems, or the adoption of multicomponent DIIS algorithms that construct combined error functions across all components,^68^ is expected to eliminate this iteration overhead.

Furthermore, we derive analytic energy gradients with respect to both classical nuclear coordinates and quantum nuclear expectation positions. The gradient expressions build upon conventional GAPW formulations,^61,62^ with additional terms that account for quantum nuclei. These terms primarily arise from the nuclear short-range external potential energy and the global Hartree energy involving nuclear soft densities. The complete gradient expressions are provided in the Supporting Information. This development enables direct geometry optimization, transition state search, and molecular dynamics simulations with NQEs inherently included within the periodic CNEO framework.

## 3 Computational Details

### 3.1 Method Implementation and Computational Setup

The CNEO–DFT method with periodic boundary conditions was implemented in the CP2K package^63,69^ and applied to a prototypical system: hydrogen chemisorption on the Pt(111) surface. In this study, hydrogen nuclei were treated quantum mechanically, while platinum atoms were treated classically as NQEs are typically negligible for heavy elements. For comparison, conventional DFT calculations were also performed using CP2K. All calculations utilized the revised Perdew–Burke–Ernzerhof (revPBE) electronic exchange–correlation functional^70,71^ with DFT-D3 dispersion corrections^72^ and Becke-Johnson damping.^73^ The interactions between electrons and quantum nuclei were treated at the mean-field level via Coulomb potentials, without incorporating any explicit epc functional. This choice is supported by previous studies,^53,85^ which have shown that existing epc functionals have a negligible impact on the overall accuracy of vibrational spectra and reaction rate constants. Nevertheless, the implementation and detailed benchmarking of epc functionals will be pursued in future work.

For hydrogen atoms, an all-electron approach was employed for the electronic part using the Ahlrichs def2-TZVP basis set,^74^ while the PB4-D basis set^75^ was used for the quantum nuclear part in CNEO–DFT calculations. The nuclear basis functions were centered at the target nuclear expectation position of each quantum nucleus. Platinum atoms were treated using the Goedecker-Teter-Hutter (GTH) pseudopotential^76^ in combination with the TZVP-MOLOPT electronic basis set.^77^ The GAPW method was applied exclusively to hydrogen atoms, while the Gaussian plane wave (GPW) approach^78–80^ was used for platinum atoms. All calculations were carried out within a spin-unpolarized framework.

The multigrid plane wave density fitting basis employed an energy cutoff of 400 Ry, and the maximum angular momentum for multipole expansions of compensating charges was set to four. For electronic SCF calculations, the standard diagonalization algorithm was employed with Broyden mixing (mixing parameter α = 0.2) and Fermi–Dirac smearing at an electronic temperature of 300 K to accommodate the metallic character of the platinum surface. For CNEO nuclear equations, the position constraint was deemed satisfied when the expectation position deviation from the basis center was less than 10^–10^ bohr. The overall SCF procedure was considered converged when the maximum electronic density matrix change between consecutive iterations did not exceed 10^–7^.

### 3.2 System Setup and Potential Energy Surface Construction

The Pt(111) surface was modeled using a seven-layer slab with a 20 Å vacuum region between periodic images to minimize spurious interactions between slabs. The cell dimensions were fixed using the experimental lattice constant of 3.9239 Å.^81^ At the first step, geometry optimization of the pristine surface was performed, in which the top five layers were allowed to relax while the bottom two layers were constrained to their bulk positions. This optimization employed a Γ-centered *k*-points mesh of 15 × 15 × 1 and was terminated when the maximum force fell below 2 × 10^–4^ hartree/bohr.

The optimized Pt slab was subsequently expanded to a 3 × 3 supercell, and one hydrogen atom was introduced, corresponding to a surface coverage of 1/9. This low coverage results in large separation between periodic images of the adsorbate and negligible hydrogen–hydrogen interactions. For all calculations involving hydrogen adsorption, the Pt positions were kept fixed at their optimized values to simplify the system and focus on the hydrogen adsorption behavior.

To construct the two-dimensional potential energy surface (PES) for hydrogen adsorption, we sampled a 32 × 32 equidistant grid spanning the 1 × 1 surface unit cell. At each (*x*, *y*) grid point, the hydrogen-surface distance (*z*-coordinate) was optimized until the perpendicular force component was less than 2 × 10^–4^ hartree/bohr, yielding the effective two-dimensional PES^82^

27 $$V\left(x,y\right)=\min_{z}E\left(x,y,z\right)$$

For each optimized geometry, we sampled four additional configurations with small displacements (±0.005 Å) along the *z*-direction to numerically determine the perpendicular vibrational frequencies. The Hessian was computed using a four-point central finite difference scheme applied to analytic gradients. For conventional DFT calculations, these frequencies were used to compute harmonic ZPE corrections to the effective PES^82^

28 $$V_{ZPE}\left(x,y\right)=V\left(x,y\right)+\frac{1}{2}\hbar \omega \left(x,y\right)$$

In contrast, for CNEO–DFT calculations, the ZPE is inherently incorporated in the total energy expression, so no such correction is needed. Nevertheless, harmonic frequencies were still computed with CNEO–DFT for direct comparison with conventional DFT results. A Γ-centered 5 × 5 × 1 *k*-points mesh was used for these calculations. Additional details, including comprehensive convergence tests with respect to the number of platinum layers and *k*-points sampling, are presented in the Supporting Information.

### 3.3 Entropy Calculation

We directly followed the work by García-Diéguez et al.^82^ to compute classical differential entropies associated with the two-dimensional in-plane motion of hydrogen atoms adsorbed on the surface, using the PESs constructed. The differential entropy was calculated using

29 $$S_{xy}=k_{B}\left[\ln\left(\frac{\alpha }{\Lambda_{th}^{2}\theta }\right)+\frac{\beta }{\alpha }+1\right]$$

where $\Lambda_{th}=h/\sqrt{2\pi mk_{B}T}$ is the thermal wavelength, θ is the hydrogen coverage, α is the effective area defined as

$$\alpha =\iint_{unitcell}\exp\left[-\frac{V\left(x,y\right)}{k_{B}T}\right]dxdy$$ 30

and the quantity β is given by

31 $$\beta =\iint_{unitcell}\frac{V\left(x,y\right)}{k_{B}T}\exp\left[-\frac{V\left(x,y\right)}{k_{B}T}\right]dxdy$$

The entropies from conventional DFT, ZPE-corrected DFT, and CNEO–DFT were evaluated by applying the classical PES, the ZPE-corrected surface *V*_ZPE_, and the CNEO–DFT effective energy surface, respectively. For additional comparison, the ideal-gas case was also computed by setting *V* = 0.

For the quantum reference based on quantum partition functions and quantum differential entropy, the 32 × 32 ZPE-corrected DFT energy surface was first interpolated to a finer 64 × 64 grid using cubic spline interpolation. Subsequently, the two-dimensional nuclear Schrödinger equation was solved using a plane-wave basis corresponding to a real-space FFT grid of size 32 × 32. The Brillouin zone for this two-dimensional periodic quantum system was sampled using a Γ-centered 33 × 33 equidistant *k*-points mesh. The mathematical formalism and detailed numerical procedures for these quantum calculations are provided in the Supporting Information.

## 4 Results and Discussion

### 4.1 Hydrogen Adsorption Sites

Figure 2 illustrates the Pt(111) surface and the key high-symmetry adsorption sites, which correspond to the most commonly observed local minima for hydrogen adsorption on close-packed metal surfaces. The atop site (red circles) represents adsorption directly above a surface Pt atom. The fcc hollow site (blue hollow hexagon) is located above a third-layer Pt atom, while the hcp hollow site (green solid hexagon) lies above a second-layer Pt atom. The dashed black lines indicate a surface unit cell.

**Figure 2:**
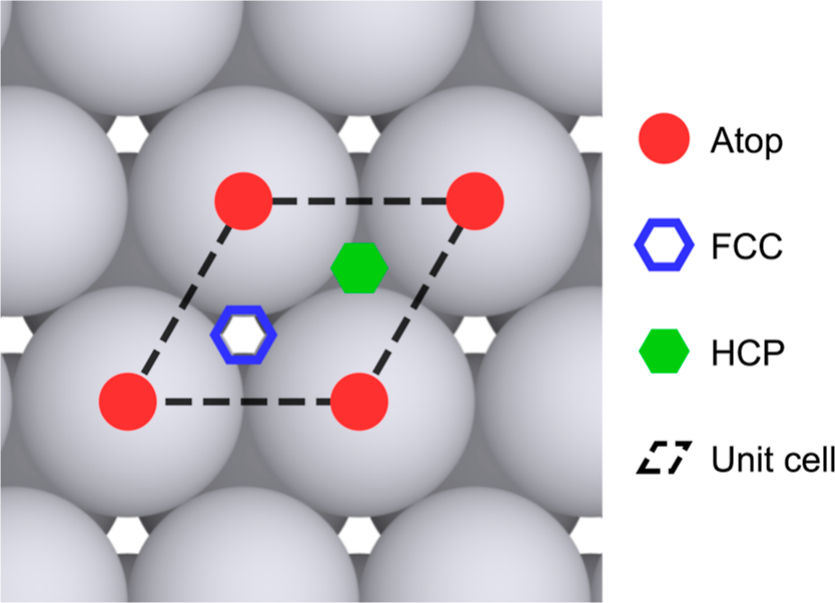
Top view of the Pt(111) surface with high-symmetry hydrogen adsorption sites: atop (red circles), fcc hollow (blue hollow hexagon), and hcp hollow (green solid hexagon). The dashed black lines indicate a surface unit cell.

Figure 3 shows the two-dimensional energy surfaces for hydrogen adsorption on the Pt(111) surface, as predicted by conventional DFT, ZPE-corrected DFT, and CNEO–DFT, evaluated within a surface unit cell. Conventional DFT calculations, which neglect NQEs, predict the atop site as the most energetically favorable adsorption position. In contrast, CNEO–DFT, which treats the proton quantum mechanically, shifts the adsorption preference to the fcc hollow site. A similar shift is observed when ZPE corrections along the *z*-direction are applied to the DFT energy surface, which is consistent with the findings reported in ref 82.

**Figure 3:**
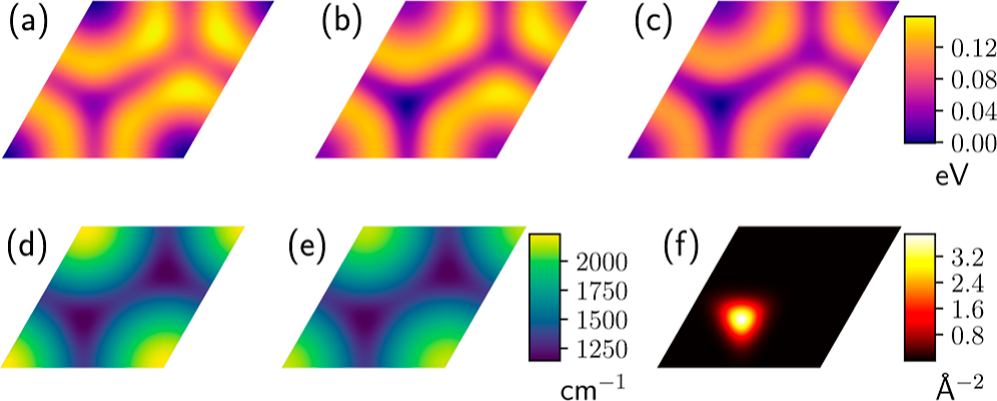
Comparison of hydrogen adsorption on Pt(111) from different computational approaches. (a) Conventional DFT potential energy surface showing the atop site as the most stable configuration. (b) ZPE-corrected DFT potential energy surface revealing the fcc hollow site as the most stable configuration. (c) CNEO–DFT potential energy surface also preferring the fcc hollow site. (d) Perpendicular motion harmonic frequencies from DFT across the surface unit cell, showing significantly higher frequencies at the atop site. (e) Perpendicular motion harmonic frequencies from CNEO–DFT, exhibiting systematically lower frequencies as NQEs are already incorporated in the CNEO treatment. (f) Quantum nuclear ground state density calculated from the ZPE-corrected DFT potential energy surface, demonstrating nuclear delocalization effects. The energy surfaces span one unit cell of the Pt(111) surface and energies are given relative to the respective global minimum.

The underlying reason for this shift in site preference can be understood by examining the perpendicular vibrational frequencies across the surface (Figure 3d). At the atop site, the Pt–H bond exhibits a substantially higher stretching frequency (approximately 2200 cm^–1^) compared to the hollow sites (approximately 1100 cm^–1^), resulting in a much higher ZPE. This difference arises from the distinct bonding environments: at the atop site, hydrogen forms a stiffer, directional bond with a single Pt atom, while at the hollow sites, the bonding is softer and distributed among three Pt atoms. The higher ZPE at the atop site, when added to the electronic energy, makes the atop site less favorable than the fcc hollow site. This ZPE effect is naturally captured in the CNEO–DFT results, where the quantum nature of the proton is explicitly incorporated in the multicomponent self-consistent field procedure.

As a benchmark reference, we also computed the quantum nuclear ground-state density by numerically solving the 2D quantum Schrödinger equation using the ZPE-corrected DFT potential energy surface (Figure 3f). It is observed that the resulting density is mostly centered at the fcc hollow site, confirming it as the quantum mechanically preferred adsorption position. Furthermore, the spatial distribution of the reference density reveals notable nuclear delocalization, with the proton density delocalized within the fcc hollow region. This shift in site preference underscores the critical role of NQEs in accurately determining the energetics of hydrogen adsorption and highlights the importance of quantum mechanical treatment when studying hydrogen adsorption on metal surfaces.

### 4.2 Hydrogen In-Plane Motion Entropy

To further quantify the impact of NQEs on the thermodynamic properties of adsorbed hydrogen, we calculated the differential entropy associated with the in-plane motion of hydrogen on the Pt(111) surface. This analysis provides insight into the mobility of the adsorbed hydrogen and its dependence on temperature and surface coverage.^82^

Figure 4 shows the temperature dependence of the differential entropy calculated using various approaches. As expected, all methods show a decrease in entropy with decreasing temperature. The 2D ideal-gas model, which assumes a flat PES, performs the worst, with particularly large deviations at low temperatures. Among the remaining methods, the entropy from conventional DFT shows the largest deviation from the quantum reference, especially in the low-temperature regime. The inclusion of ZPE corrections improves the agreement but still exhibits notable discrepancies. Remarkably, the CNEO–DFT results provide the closest match to the quantum reference across the entire investigated temperature range, especially in the catalytically relevant regime of 500–800 K.

**Figure 4:**
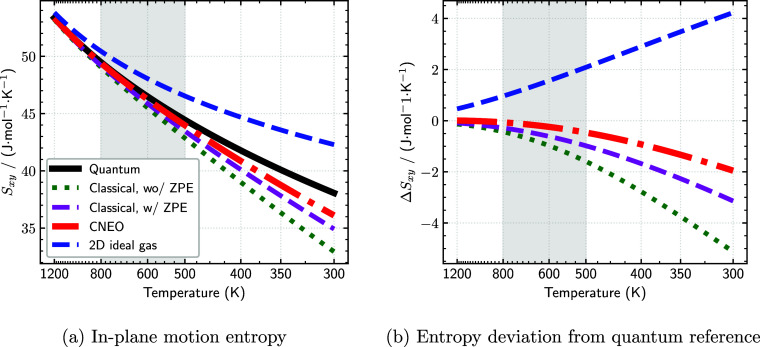
Temperature dependence of the 2D in-plane motion differential entropy calculated using different approaches. (a) Comparison of entropy values derived from classical integrations over conventional DFT (green), ZPE-corrected DFT (magenta), and CNEO–DFT (red) energy surfaces, alongside the quantum reference calculation (black) and ideal gas comparison (blue). (b) Difference between the classical integration approaches and the quantum reference. The shaded area indicates the temperature range relevant for catalytic applications (500–800 K).

The enhanced accuracy of CNEO–DFT in predicting the differential entropy stems from two key factors. First, CNEO–DFT correctly identifies the fcc hollow site as the energetically preferred adsorption site, consistent with the quantum reference as well as ZPE-corrected DFT. Second, and more subtly, the CNEO–DFT energy surface exhibits lower barriers for hydrogen diffusion between neighboring adsorption sites compared to the ZPE-corrected DFT surface (Figure 3b and c). This difference arises because CNEO–DFT treats the proton quantum mechanically in all three dimensions, naturally incorporating both ZPE along the *z*-direction and shallow tunneling effects in the *xy* plane. In contrast, the ZPE-corrected DFT approach only accounts for ZPE along the *z*-direction. As a result, even classical statistical calculations performed on the CNEO–DFT energy surface can effectively capture the key aspects of quantum nuclear behavior that would otherwise require more complex quantum mechanical treatments.

At low temperatures, all classical approaches exhibit larger deviations from the quantum reference (Figure 4b). This discrepancy arises from the fundamental limitation of classical statistical mechanics in capturing energy-level quantization effects, which becomes increasingly important as temperature decreases. Classical entropy expressions can even yield unphysical negative values as temperature approaches zero. Nevertheless, for most practical applications in catalysis and surface chemistry, where temperatures typically exceed 300 K, the CNEO–DFT approach provides an excellent approximation to the fully quantum mechanical result.

Additionally, we investigated the dependence of in-plane motion entropy on the surface coverage of hydrogen. The coverage dependence of the differential entropy at 598 K is presented in Figure 5. Following the work by Borodin et al.,^83^ we assumed noninteracting hydrogen atoms and used the same energy surfaces computed earlier for all coverage values. This simplification is appropriate in the dilute coverage regime, where adsorbate–adsorbate interactions are minimal. As expected, all entropy curves show the characteristic −*k*_B_ ln θ dependence on coverage, resulting in parallel trends.

**Figure 5:**
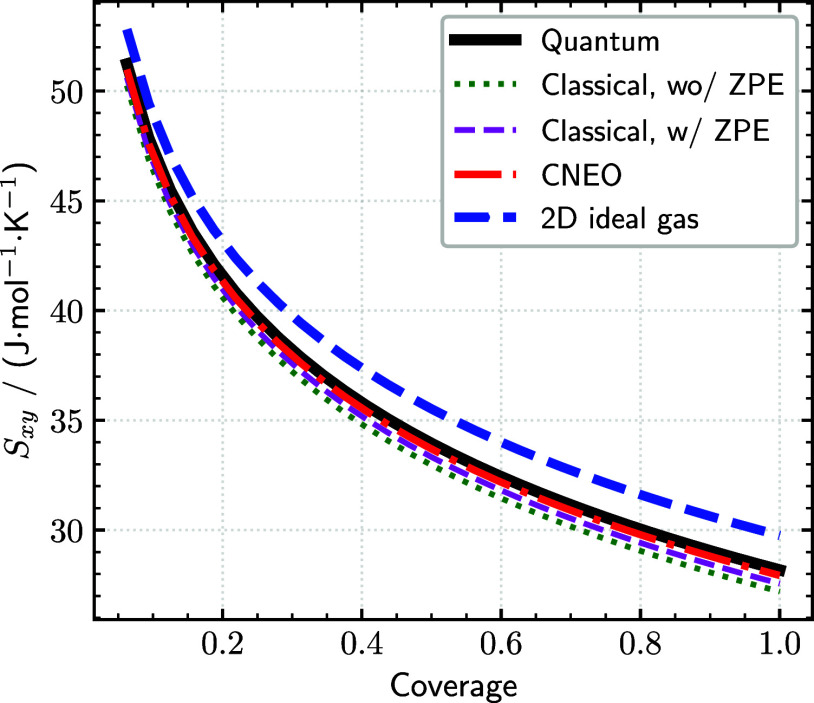
Coverage-dependent differential entropy at 598 K for hydrogen on Pt(111) within the noninteracting hydrogen approximation. Comparison between quantum reference results (black), conventional DFT (green), ZPE-corrected DFT (magenta), CNEO–DFT (red), and ideal gas (blue). All approaches show the expected −*k*_*B*_ ln θ dependence on coverage θ, but with different absolute values.

At 598 K, a temperature highly relevant to catalytic processes, the CNEO–DFT predictions show excellent agreement with the quantum reference, with deviations of less than 0.3 J ·mol^–1^·K^–1^. Conventional DFT results exhibit slightly larger discrepancies, although now they are also close to the quantum reference at this elevated temperature. These observations are expected, as 598 K is sufficiently high for hydrogen motion to be predominantly classical, although quantum effects remain non-negligible.

It is worth noting that ref 83 reported more substantial differences between classical and quantum entropy values even at 598 K. This discrepancy may arise from the distinction between the differential entropy (∂*S*/∂*N*), as calculated in this work following ref 82, and the entropy per particle (*S*/*N*) reported in ref 83. These two quantities typically differ by *k*_B_, as further discussed in the Supporting Information.

## 5 Conclusions

We have developed CNEO–DFT with periodic boundary conditions within the GAPW framework of the CP2K software package. This implementation enables the quantum mechanical treatment of nuclei in extended systems while maintaining computational efficiency comparable to conventional DFT calculations.

The CNEO–DFT implementation employs the distinguishable-particle approximation, which is physically justified by the highly localized nature of quantum nuclear wave functions and the minimal overlap between neighboring nuclei. This approximation allows each quantum nucleus to be described by its own single-particle density and avoids the computational complexity associated with nuclear Bloch states. Periodicity is preserved through the collective nuclear distribution. Position constraints are applied during the SCF procedure to ensure that the nuclear expectation positions match classical coordinates. This framework enables the construction of effective energy surfaces with NQEs incorporated, while preserving well-defined geometric information essential for chemical interpretation.

The GAPW spatial partitioning scheme divides the system into atomic regions and interstitial space, allowing efficient treatment of both smooth and sharply peaked electronic and quantum nuclear densities through computational strategies optimized for each domain. Compensation charges are employed to facilitate the accurate and efficient evaluation of the electrostatic energy, while nuclear self-interaction terms are naturally eliminated through a reformulated expression. A simultaneous SCF procedure is employed, alternating between nuclear and electronic updates. Analytic gradients are implemented for both classical nuclear coordinates and quantum nuclear expectation positions, supporting efficient geometry optimizations and molecular dynamics simulations. The additional computational cost relative to conventional DFT is minimal, as the nuclear-specific energy terms are evaluated within the existing GAPW infrastructure without introducing additional computationally intensive operations.

As a demonstration, we applied the developed method to hydrogen adsorption on Pt(111), our method reveals that NQEs qualitatively shift the preferred adsorption site: conventional DFT predicts the atop site as the most favorable, while CNEO–DFT identifies the fcc hollow site, consistent with the quantum reference as well as ZPE-corrected DFT. This shift is primarily driven by ZPE contributions and highlights the importance of the quantum mechanical treatment of the nuclei. Entropy analysis further shows that NQEs lower the effective barriers for hydrogen diffusion and remain significant even at catalytically relevant temperatures (500–800 K). CNEO–DFT yields differential entropy values in close agreement with the quantum reference, outperforming both conventional and ZPE-corrected DFT. This accuracy stems from CNEO–DFT’s ability to capture ZPE and shallow tunneling effects in different spatial directions.

This periodic development of CNEO–DFT opens new opportunities for investigating NQEs in extended systems with computational efficiency comparable to conventional DFT. By providing more accurate predictions of thermodynamic properties such as adsorption preferences and entropy, CNEO–DFT could offer a promising path toward generating reliable thermodynamic parameters for microkinetic models of surface reactions, especially those involving light nuclei like hydrogen. These improvements are especially relevant for catalytic processes at moderate temperatures, where NQEs remain significant yet are frequently neglected in conventional modeling approaches.

Future extensions of this framework may include the development of multicomponent correlation functionals, the application to more complex interfacial systems where proton behavior critically influences structure and reactivity, and the incorporation of NQEs into free energy calculations for surface processes and heterogeneous catalysis.

## Supplementary Material



## Notes

(1) Detailed theoretical framework including review of multicomponent DFT for molecules and comprehensive derivation of CNEO–DFT with periodic boundary conditions within the GAPW formalism; (2) complete expressions for the Ewald summation correction terms *E*_self_ and *E*_ovrl_; (3) explicit matrix elements for the coupled nuclear and electronic Kohn–Sham Hamiltonians within the GAPW framework, including the treatment of *k*-point sampling for the electronic Hamiltonian; (4) full analytic gradient expressions for both classical nuclear coordinates and quantum nuclear expectation positions; (5) mathematical formalism and numerical procedures for quantum reference calculations, including the solution of the 2D nuclear Schrödinger equation with periodic potentials and quantum statistical mechanical calculations of thermodynamic properties; (6) convergence tests with respect to *k*-point sampling density and number of platinum layers in the slab model (PDF)
