# Supplementary material for: Periodic Constrained Nuclear-Electronic Orbital Density Functional Theory for Nuclear Quantum Effects: Method Development and Application to Hydrogen Adsorption on Pt(111)
Source: J Chem Theory Comput. 2025 Aug 11;21(16):7865–77. doi: 10.1021/acs.jctc.5c00837 (PMC12503368; doi:10.1021/acs.jctc.5c00837)
Supplement: Supplementary file 1 [file ct5c00837_si_001.pdf]

Supporting Information: Periodic Constrained Nuclear-Electronic Orbital Density Functional Theory for Nuclear Quantum Effects: Method Development and Application to Hydrogen Adsorption on Pt(111)

Zehua Chen* and Yang Yang*

Theoretical Chemistry Institute and Department of Chemistry, University of Wisconsin-Madison, 1101 University Avenue, Madison, Wisconsin 53706, United States

E-mail: zchen444@wisc.edu; yyang222@wisc.edu

S1 Review of (constrained) multicomponent DFT for molecules

In conventional electronic structure methods, nuclei are treated as classical point charges that generate an external potential for electrons. However, this approximation neglects nuclear quantum effects, which can introduce significant errors in chemical systems involving light nuclei, such as hydrogen. Multicomponent density functional theory (DFT) treats both nuclei and electrons quantum mechanically, which provides a rigorous framework for describing nuclear quantum effects.

For a molecular system where certain nuclei (typically protons) require quantum mechan-

ical treatment, the total energy can be expressed as a minimization of a multicomponent density functional:

$$E = \min_{\rho^{\mathrm{e}} \to N^{\mathrm{e}}, \rho^{\mathrm{n}} \to N^{\mathrm{n}}} E_{V_{\mathrm{ext}}} [\rho^{\mathrm{e}}, \rho^{\mathrm{n}}] + E_{\mathrm{nuc}}. \tag{S1}$$

Here, $E_{V_{\mathrm{ext}}}$ represents the multicomponent density functional under the external Coulombic potential generated by the remaining classical nuclei:

$$E_{V_{\mathrm{ext}}} [\rho^{\mathrm{e}}, \rho^{\mathrm{n}}] = F_{\mathrm{HK}}^{\mathrm{mult}} [\rho^{\mathrm{e}}, \rho^{\mathrm{n}}] + \int \mathrm{d}\boldsymbol{r} V_{\mathrm{ext}}(\boldsymbol{r}) [\rho^{\mathrm{e}}(\boldsymbol{r}) - Z^{\mathrm{n}} \rho^{\mathrm{n}}(\boldsymbol{r})], \tag{S2}$$

and E_{nuc} is the classical nuclear repulsion energy. For clarity, we present the formalism with a single type of quantum nuclei carrying charge $+Z^{\mathrm{n}}$, though the extension to multiple types with different charges is straightforward. $F_{\mathrm{HK}}^{\mathrm{mult}}$ represents the universal multicomponent Hohenberg-Kohn functional, which can be defined via Levy's constrained search over multicomponent wave functions.

In the Kohn-Sham formalism of multicomponent DFT, the non-interacting multicomponent wave function is expressed as a direct product of electronic and nuclear Slater determinants (assuming fermionic quantum nuclei):

$$\Psi (\boldsymbol{r}_1^{\mathrm{e}}, \cdots, \boldsymbol{r}_{N^{\mathrm{e}}}^{\mathrm{e}}, \boldsymbol{r}_1^{\mathrm{n}}, \cdots, \boldsymbol{r}_{N^{\mathrm{n}}}^{\mathrm{n}}) = \Phi^{\mathrm{e}} (\boldsymbol{r}_1^{\mathrm{e}}, \cdots, \boldsymbol{r}_{N^{\mathrm{e}}}^{\mathrm{e}}) \Phi^{\mathrm{n}} (\boldsymbol{r}_1^{\mathrm{n}}, \cdots, \boldsymbol{r}_{N^{\mathrm{n}}}^{\mathrm{n}}). \tag{S3}$$

This product form enables a decomposition of the energy functional:

$$\begin{aligned} E_{V_{\mathrm{ext}}} [\rho^{\mathrm{e}}, \rho^{\mathrm{n}}] = {} & T_{\mathrm{s}}^{\mathrm{e}}[\rho^{\mathrm{e}}] + T_{\mathrm{s}}^{\mathrm{n}} [\rho^{\mathrm{n}}] + E_{\mathrm{ext}}^{\mathrm{e}}[\rho^{\mathrm{e}}] + E_{\mathrm{ext}}^{\mathrm{n}}[\rho^{\mathrm{n}}] \\ & + E_{\mathrm{H}} [\rho^{\mathrm{e}} - Z^{\mathrm{n}} \rho^{\mathrm{n}}] + E_{\mathrm{xc}}^{\mathrm{e}}[\rho^{\mathrm{e}}] + E_{\mathrm{xc}}^{\mathrm{n}} [\rho^{\mathrm{n}}] + E_{\mathrm{c}} [\rho^{\mathrm{e}}, \rho^{\mathrm{n}}], \end{aligned} \tag{S4}$$

where $T_{\mathrm{s}}^{\mathrm{e}}$ and $T_{\mathrm{s}}^{\mathrm{n}}$ represent the non-interacting kinetic energies for electrons and quantum nuclei, respectively. The interaction with the external potential is given by:

$$E_{\mathrm{ext}}^{\mathrm{e}}[\rho^{\mathrm{e}}] = \int \mathrm{d}\boldsymbol{r} \rho^{\mathrm{e}}(\boldsymbol{r}) V_{\mathrm{ext}}(\boldsymbol{r}), \tag{S5}$$

$$E_{\rm ext}^{\rm n}[\rho^{\rm n}] = -Z^{\rm n} \int {\rm d}\boldsymbol{r} \rho^{\rm n}(\boldsymbol{r}) V_{\rm ext}(\boldsymbol{r}). \tag{S6}$$

The Hartree energy $E_{\rm H}$ accounts for all Coulombic interactions:

$$\begin{aligned} E_{\rm H}\left[\rho^{\rm e} - Z^{\rm n}\rho^{\rm n}\right] = &\frac{1}{2}\int {\rm d}\boldsymbol{r} \int {\rm d}\boldsymbol{r}' \frac{\rho^{\rm e}(\boldsymbol{r})\rho^{\rm e}(\boldsymbol{r}')}{|\boldsymbol{r}-\boldsymbol{r}'|} + \frac{(Z^{\rm n})^2}{2}\int {\rm d}\boldsymbol{r} \int {\rm d}\boldsymbol{r}' \frac{\rho^{\rm n}(\boldsymbol{r})\rho^{\rm n}(\boldsymbol{r}')}{|\boldsymbol{r}-\boldsymbol{r}'|} \\ &- Z^{\rm n}\int {\rm d}\boldsymbol{r} \int {\rm d}\boldsymbol{r}' \frac{\rho^{\rm e}(\boldsymbol{r})\rho^{\rm n}(\boldsymbol{r}')}{|\boldsymbol{r}-\boldsymbol{r}'|}, \end{aligned} \tag{S7}$$

which can be rewritten in terms of the total charge density:

$$E_{\rm H}\left[\rho^{\rm e} - Z^{\rm n}\rho^{\rm n}\right] = \frac{1}{2}\int {\rm d}\boldsymbol{r} \int {\rm d}\boldsymbol{r}' \frac{\left[\rho^{\rm e}(\boldsymbol{r}) - Z^{\rm n}\rho^{\rm n}(\boldsymbol{r})\right]\left[\rho^{\rm e}(\boldsymbol{r}') - Z^{\rm n}\rho^{\rm n}(\boldsymbol{r}')\right]}{|\boldsymbol{r}-\boldsymbol{r}'|}. \tag{S8}$$

The exchange-correlation terms $E_{\rm xc}^{\rm e}$ and $E_{\rm xc}^{\rm n}$ represent the single-component exchange-correlation energies for electrons and quantum nuclei, respectively. These are defined as the residual quantities from the Hohenberg-Kohn universal functional after accounting for the non-interacting kinetic energy and Hartree energy in the Kohn-Sham system:

$$E_{\rm xc}^{\rm e}[\rho^{\rm e}] = F_{\rm HK}^{\rm e}[\rho^{\rm e}] - \left\{T_{\rm s}^{\rm e}[\rho^{\rm e}] + \frac{1}{2}\int {\rm d}\boldsymbol{r} \int {\rm d}\boldsymbol{r}' \frac{\rho^{\rm e}(\boldsymbol{r})\rho^{\rm e}(\boldsymbol{r}')}{|\boldsymbol{r}-\boldsymbol{r}'|}\right\}, \tag{S9}$$

$$E_{\rm xc}^{\rm n}\left[\rho^{\rm n}\right] = F_{\rm HK}^{\rm n}\left[\rho^{\rm n}\right] - \left\{T_{\rm s}^{\rm n}\left[\rho^{\rm n}\right] + \frac{(Z^{\rm n})^2}{2}\int {\rm d}\boldsymbol{r} \int {\rm d}\boldsymbol{r}' \frac{\rho^{\rm n}(\boldsymbol{r})\rho^{\rm n}(\boldsymbol{r}')}{|\boldsymbol{r}-\boldsymbol{r}'|}\right\}. \tag{S10}$$

Finally, $E_{\rm c}$ represents the multicomponent correlation energy—the difference between the full multicomponent Hohenberg-Kohn universal functional and the sum of individual single-component contributions plus mean-field Coulomb interactions:

$$E_{\rm c}\left[\rho^{\rm e},\rho^{\rm n}\right] = F_{\rm HK}^{\rm mult}\left[\rho^{\rm e},\rho^{\rm n}\right] - \left\{F_{\rm HK}^{\rm e}[\rho^{\rm e}] + F_{\rm HK}^{\rm n}\left[\rho^{\rm n}\right] - Z^{\rm n}\int {\rm d}\boldsymbol{r} \int {\rm d}\boldsymbol{r}' \frac{\rho^{\rm e}(\boldsymbol{r})\rho^{\rm n}(\boldsymbol{r}')}{|\boldsymbol{r}-\boldsymbol{r}'|}\right\}. \tag{S11}$$

The coupled Kohn-Sham equations for electrons and quantum nuclei are:

$$\left[-\frac{\hbar^2}{2m_{\rm e}}\nabla^2 + V_{\rm eff}^{\rm e}(\boldsymbol{r})\right]\psi_i^{\rm e}(\boldsymbol{r}) = \varepsilon_i^{\rm e}\psi_i^{\rm e}(\boldsymbol{r}), \tag{S12}$$

$$\left[-\frac{\hbar^2}{2m_{\mathrm{n}}}\nabla^2 + V_{\mathrm{eff}}^{\mathrm{n}}(\boldsymbol{r})\right]\psi_i^{\mathrm{n}}(\boldsymbol{r}) = \varepsilon_i^{\mathrm{n}}\psi_i^{\mathrm{n}}(\boldsymbol{r}), \tag{S13}$$

where the effective potentials contain external, Hartree, and exchange-correlation contributions:

$$V_{\mathrm{eff}}^{\mathrm{e}}(\boldsymbol{r}) = V_{\mathrm{ext}}(\boldsymbol{r}) + \int \mathrm{d}\boldsymbol{r}' \frac{\rho^{\mathrm{e}}(\boldsymbol{r}')}{|\boldsymbol{r}-\boldsymbol{r}'|} - Z^{\mathrm{n}}\int \mathrm{d}\boldsymbol{r}' \frac{\rho^{\mathrm{n}}(\boldsymbol{r}')}{|\boldsymbol{r}-\boldsymbol{r}'|} + V_{\mathrm{xc}}^{\mathrm{e}}[\rho^{\mathrm{e}}](\boldsymbol{r}) + \frac{\delta E_{\mathrm{c}}\left[\rho^{\mathrm{e}},\rho^{\mathrm{n}}\right]}{\delta\rho^{\mathrm{e}}(\boldsymbol{r})}, \tag{S14}$$

$$V_{\mathrm{eff}}^{\mathrm{n}}(\boldsymbol{r}) = -Z^{\mathrm{n}}V_{\mathrm{ext}}(\boldsymbol{r}) - Z^{\mathrm{n}}\int \mathrm{d}\boldsymbol{r}' \frac{\rho^{\mathrm{e}}(\boldsymbol{r}')}{|\boldsymbol{r}-\boldsymbol{r}'|} + (Z^{\mathrm{n}})^2\int \mathrm{d}\boldsymbol{r}' \frac{\rho^{\mathrm{n}}(\boldsymbol{r}')}{|\boldsymbol{r}-\boldsymbol{r}'|} + V_{\mathrm{xc}}^{\mathrm{n}}\left[\rho^{\mathrm{n}}\right](\boldsymbol{r}) + \frac{\delta E_{\mathrm{c}}\left[\rho^{\mathrm{e}},\rho^{\mathrm{n}}\right]}{\delta\rho^{\mathrm{n}}(\boldsymbol{r})}. \tag{S15}$$

S1.1 Distinguishable-particle approximation

A significant computational simplification arises from the distinguishable-particle approximation. Nuclear wave functions are typically extremely localized in space, making the overlap between neighboring quantum nuclei negligible. This localization enables us to replace the Slater determinant for N^{n} fermionic quantum nuclei with a simpler Hartree product of N^{n} single-particle states:

$$\Psi\left(\boldsymbol{r}_1^{\mathrm{e}},\cdots,\boldsymbol{r}_{N^{\mathrm{e}}}^{\mathrm{e}},\boldsymbol{r}_1^{\mathrm{n}},\cdots,\boldsymbol{r}_{N^{\mathrm{n}}}^{\mathrm{n}}\right) = \Phi^{\mathrm{e}}\left(\boldsymbol{r}_1^{\mathrm{e}},\cdots,\boldsymbol{r}_{N^{\mathrm{e}}}^{\mathrm{e}}\right)\Phi_1^{\mathrm{n}}\left(\boldsymbol{r}_1^{\mathrm{n}}\right)\Phi_2^{\mathrm{n}}\left(\boldsymbol{r}_2^{\mathrm{n}}\right)\cdots\Phi_{N^{\mathrm{n}}}^{\mathrm{n}}\left(\boldsymbol{r}_{N^{\mathrm{n}}}^{\mathrm{n}}\right). \tag{S16}$$

Since each quantum nucleus is treated as a single particle, its self-Coulomb and self-exchange effects cancel exactly, and there is no self-correlation. The multicomponent energy functional thus simplifies to:

$$\begin{aligned} E_{V_{\mathrm{ext}}}\left[\rho^{\mathrm{e}},\rho_1^{\mathrm{n}},\rho_2^{\mathrm{n}},\cdots,\rho_{N^{\mathrm{n}}}^{\mathrm{n}}\right] = T_{\mathrm{s}}^{\mathrm{e}}[\rho^{\mathrm{e}}] + \sum_{a=1}^{N^{\mathrm{n}}} T_{\mathrm{s}}^{\mathrm{n},a}\left[\rho_a^{\mathrm{n}}\right] + \int \mathrm{d}\boldsymbol{r} V_{\mathrm{ext}}(\boldsymbol{r})\left[\rho^{\mathrm{e}}(\boldsymbol{r}) - \sum_{a=1}^{N^{\mathrm{n}}} Z_a\rho_a^{\mathrm{n}}(\boldsymbol{r})\right] \\ + E_{\mathrm{H}}\left[\rho^{\mathrm{e}},\{\rho_a^{\mathrm{n}}\}\right] + E_{\mathrm{xc}}^{\mathrm{e}}[\rho^{\mathrm{e}}] + E_{\mathrm{c}}\left[\rho^{\mathrm{e}},\rho_1^{\mathrm{n}},\rho_2^{\mathrm{n}},\cdots,\rho_{N^{\mathrm{n}}}^{\mathrm{n}}\right], \end{aligned} \tag{S17}$$

where Z_a is the charge for a-th quantum nucleus. This formulation naturally accommodates multiple types of quantum nuclei. The total Hartree energy can be evaluated by removing the nuclear single-particle self-Coulomb energies from the Hartree energy of the total charge density:

$$E_{\mathrm{H}}\left[\rho^{\mathrm{e}},\{\rho_a^{\mathrm{n}}\}\right]=E_{\mathrm{H}}\left[\rho^{\mathrm{e}}-\sum_a Z_a\rho_a^{\mathrm{n}}\right]-\sum_a E_{\mathrm{H}}\left[Z_a\rho_a^{\mathrm{n}}\right] \tag{S18}$$

$$\begin{aligned}&=\frac{1}{2}\int \mathrm{d}\boldsymbol{r}\int \mathrm{d}\boldsymbol{r}'\frac{\left[\rho^{\mathrm{e}}(\boldsymbol{r})-\sum_a Z_a\rho_a^{\mathrm{n}}(\boldsymbol{r})\right]\left[\rho^{\mathrm{e}}(\boldsymbol{r}')-\sum_a Z_a\rho_a^{\mathrm{n}}(\boldsymbol{r}')\right]}{|\boldsymbol{r}-\boldsymbol{r}'|}\\&\quad-\frac{1}{2}\sum_a Z_a^2\int \mathrm{d}\boldsymbol{r}\int \mathrm{d}\boldsymbol{r}'\frac{\rho_a^{\mathrm{n}}(\boldsymbol{r})\rho_a^{\mathrm{n}}(\boldsymbol{r}')}{|\boldsymbol{r}-\boldsymbol{r}'|}\end{aligned} \tag{S19}$$

$$\begin{aligned}&=\frac{1}{2}\int \mathrm{d}\boldsymbol{r}\int \mathrm{d}\boldsymbol{r}'\frac{\rho^{\mathrm{e}}(\boldsymbol{r})\rho^{\mathrm{e}}(\boldsymbol{r}')}{|\boldsymbol{r}-\boldsymbol{r}'|}-\sum_a Z_a\int \mathrm{d}\boldsymbol{r}\int \mathrm{d}\boldsymbol{r}'\frac{\rho^{\mathrm{e}}(\boldsymbol{r})\rho_a^{\mathrm{n}}(\boldsymbol{r}')}{|\boldsymbol{r}-\boldsymbol{r}'|}\\&\quad+\frac{1}{2}\sum_{a\neq b} Z_aZ_b\int \mathrm{d}\boldsymbol{r}\int \mathrm{d}\boldsymbol{r}'\frac{\rho_a^{\mathrm{n}}(\boldsymbol{r})\rho_b^{\mathrm{n}}(\boldsymbol{r}')}{|\boldsymbol{r}-\boldsymbol{r}'|}.\end{aligned} \tag{S20}$$

The Kohn-Sham effective potential for a-th quantum nucleus consequently becomes:

$$V_{\mathrm{eff}}^{\mathrm{n},a}(\boldsymbol{r})=-Z_aV_{\mathrm{ext}}(\boldsymbol{r})-Z_a\int \mathrm{d}\boldsymbol{r}'\frac{\rho^{\mathrm{e}}(\boldsymbol{r}')}{|\boldsymbol{r}-\boldsymbol{r}'|}+Z_a\sum_{b\neq a}Z_b\int \mathrm{d}\boldsymbol{r}'\frac{\rho_b^{\mathrm{n}}(\boldsymbol{r}')}{|\boldsymbol{r}-\boldsymbol{r}'|}+\frac{\delta E_{\mathrm{c}}\left[\rho^{\mathrm{e}},\rho_1^{\mathrm{n}},\rho_2^{\mathrm{n}},\cdots,\rho_{N^{\mathrm{n}}}^{\mathrm{n}}\right]}{\delta\rho_a^{\mathrm{n}}(\boldsymbol{r})}. \tag{S21}$$

S1.2 Nuclear expectation position constraints

Constrained nuclear-electronic-orbital DFT (CNEO-DFT) introduces an additional innovation by imposing position constraints on quantum nuclei:

$$\int \mathrm{d}\boldsymbol{r}\rho_a^{\mathrm{n}}(\boldsymbol{r})\boldsymbol{r}=\boldsymbol{R}_a, \tag{S22}$$

where $\boldsymbol{R}_a$ represents the desired expectation position for the a-th quantum nucleus. This constraint is implemented using the Lagrange multiplier method by defining the Lagrangian:

$$\mathcal{L} = E_{V_{\text{ext}}}\left[\rho^{\text{e}}, \{\rho_a^{\text{n}}\}\right] - \sum_i \varepsilon_i^{\text{e}} \left(\langle\psi_i^{\text{e}}|\psi_i^{\text{e}}\rangle - 1\right) - \sum_a \sum_i \varepsilon_i^{\text{n},a} \left(\langle\psi_i^{\text{n},a}|\psi_i^{\text{n},a}\rangle - 1\right) + \boldsymbol{f}_a \cdot \int \text{d}\boldsymbol{r} \rho_a^{\text{n}}(\boldsymbol{r})(\boldsymbol{r} - \boldsymbol{R}_a). \tag{S23}$$

This approach leads to the constrained DFT (CDFT) Kohn-Sham equation for quantum nuclei:

$$\left[-\frac{\hbar^2}{2m_{\text{n}}}\nabla^2 + V_{\text{eff}}^{\text{n},a}(\boldsymbol{r}) + \boldsymbol{f}_a \cdot (\boldsymbol{r} - \boldsymbol{R}_a)\right] \psi_i^{\text{n},a}(\boldsymbol{r}) = \varepsilon_i^{\text{n},a} \psi_i^{\text{n},a}(\boldsymbol{r}), \tag{S24}$$

where the Lagrange multiplier $\boldsymbol{f}_a$ must be iteratively optimized to ensure that the singly-occupied ground state nuclear orbital $\psi_{\text{g.s.}}^{\text{n},a}$ satisfies the position constraint. The CNEO-DFT approach maintains well-defined molecular structures while incorporating nuclear quantum effects into the potential energy landscape.

S2 CNEO-DFT with periodic boundary conditions

Extending constrained nuclear-electronic orbital density functional theory (CNEO-DFT) to periodic systems introduces several conceptual and computational challenges. In this section, we present a comprehensive theoretical framework for implementing CNEO-DFT under periodic boundary conditions (PBC). Our approach addresses: (1) the representation of quantum nuclei in extended systems, (2) the evaluation of long-range electrostatic interactions, (3) the decomposition of charge densities within the Gaussian-augmented plane wave method, (4) the formulation of the total energy expression, (5) the coupled nuclear-electronic Kohn-Sham equations, (6) the self-consistent field procedure for solving these equations, and (7) the derivation of analytic energy gradients for geometry optimization and molecular dynamics. This integrated framework enables efficient quantum mechanical treatment of selected nuclei in periodic systems while maintaining computational tractability.

S2.1 Representation of quantum nuclei in periodic systems

In conventional periodic electronic structure theory, Kohn-Sham orbitals form Bloch states characterized by a wave vector $\boldsymbol{k}$:

$$\psi^{\mathrm{e}}_{i\boldsymbol{k}}(\boldsymbol{r}) = \mathrm{e}^{\mathrm{i}\boldsymbol{k}\cdot\boldsymbol{r}} u^{\mathrm{e}}_{i\boldsymbol{k}}(\boldsymbol{r}), \tag{S25}$$

where $u^{\mathrm{e}}_{i\boldsymbol{k}}$ is a periodic function that satisfies:

$$u^{\mathrm{e}}_{i\boldsymbol{k}}(\boldsymbol{r} + n_1\boldsymbol{a}_1 + n_2\boldsymbol{a}_2 + n_3\boldsymbol{a}_3) \equiv u^{\mathrm{e}}_{i\boldsymbol{k}}(\boldsymbol{r} + \boldsymbol{T}_{\boldsymbol{n}}) = u^{\mathrm{e}}_{i\boldsymbol{k}}(\boldsymbol{r}), \quad \forall n_1, n_2, n_3 \in \mathbb{Z}, \tag{S26}$$

with $\boldsymbol{n} = (n_1, n_2, n_3)$ and $\boldsymbol{T}_{\boldsymbol{n}}$ denoting a lattice translation vector. The electron density is constructed by integrating over the first Brillouin zone (BZ), with appropriate occupation numbers:

$$\rho^{\mathrm{e}}(\boldsymbol{r}) = \frac{1}{\Omega_{\mathrm{BZ}}} \int_{\Omega_{\mathrm{BZ}}} \mathrm{d}\boldsymbol{k} \sum_i f^{\mathrm{e}}_{i\boldsymbol{k}} \left| u^{\mathrm{e}}_{i\boldsymbol{k}}(\boldsymbol{r}) \right|^2. \tag{S27}$$

A conceptual challenge arises when considering how to treat quantum nuclei within this framework. One might initially expect that nuclear orbitals should also form Bloch states, which would make it difficult to define expectation positions that can have values in every unit cell. Indeed, Wannier functions can be used in this case to help define unambiguous positions for quantum nuclei. However, the extreme localization of nuclear wave functions provides a natural solution and the complication of nuclear Bloch states and Wannier functions can be avoided altogether.

Due to the highly localized nature of quantum nuclear densities, the dispersion of nuclear wave functions across the Brillouin zone is negligible (also common in electronic band structures for tightly-binding core electrons), we adopt the distinguishable-particle approximation even for periodic systems. Rather than using square-non-integrable nuclear Bloch states, we employ localized square-integrable single-particle Kohn-Sham orbitals for each quantum nucleus. The periodic indistinguishable nuclear density is then constructed as the

sum of infinitely many distinguishable particle densities:

$$\rho^{\mathrm{n}}(\boldsymbol{r}) = \sum_{a} \rho^{\mathrm{n}}_{a}(\boldsymbol{r}), \tag{S28}$$

where a indexes all quantum nuclei in the unit cell and all their periodic images. Again the formalism here only illustrates a single type of quantum nuclei. The extension to multiple types is also straightforward, by grouping the distinguishable particle densities of the same type to form the corresponding indistinguishable density.

While this formulation apparently leads to an infinite number of nuclear Kohn-Sham equations, the periodicity of the quantum nuclear density:

$$\rho^{\mathrm{n}}(\boldsymbol{r} + \boldsymbol{T}_{\boldsymbol{n}}) = \rho^{\mathrm{n}}(\boldsymbol{r}) \tag{S29}$$

allows us to solve the nuclear Kohn-Sham equations only for nuclei within the unit cell. Densities for all other unit cells can be obtained through simple translations:

$$\rho^{\mathrm{n}}(\boldsymbol{r}) = \sum_{a \in \text{unit cell}} \sum_{\boldsymbol{n}} \rho^{\mathrm{n}}_{a}(\boldsymbol{r} + \boldsymbol{T}_{\boldsymbol{n}}). \tag{S30}$$

This approach is analogous to how point-charge classical nuclei create a periodic charge distribution in conventional DFT calculations. The distinguishable-particle formulation provides a natural computational advantage while maintaining the proper periodicity required for extended systems.

S2.2 Electrostatic energy evaluation

A central challenge in evaluating the total energy of a periodic system is the treatment of the long-range Coulomb interactions, particularly those involving classical nuclei. For a periodic system with both classical and quantum nuclei, we must carefully account for the electrostatic interactions to obtain a well-defined, non-divergent energy per unit cell.

The external potential in our system is the Coulombic potential generated by classical nuclei:

$$V_{\mathrm{ext}}(\boldsymbol{r}) = -\sum_{A} Z_A \frac{1}{|\boldsymbol{r} - \boldsymbol{R}_A|}, \tag{S31}$$

where A indexes all classical nuclei in the unit cell and their periodic images. The classical nuclear repulsion energy per unit cell is:

$$E_{\mathrm{nuc}} = \frac{1}{2} \sum_{\substack{A,B \in \text{unit cell} \\ A \neq B}} \frac{Z_A Z_B}{|\boldsymbol{R}_A - \boldsymbol{R}_B|} + \frac{1}{2} \sum_{\boldsymbol{n} \neq (0,0,0)} \sum_{A \in \text{unit cell}} \sum_{B \in \text{unit cell}} \frac{Z_A Z_B}{|\boldsymbol{R}_A - \boldsymbol{R}_B - \boldsymbol{T}_{\boldsymbol{n}}|}. \tag{S32}$$

To evaluate this energy efficiently while avoiding divergences, we employ the Ewald summation technique,[S1] which separates Coulomb interactions into short-range and long-range components. This approach transforms the nuclear repulsion energy into:

$$E_{\mathrm{nuc}} = \frac{1}{2} \int_{\Omega_{\mathrm{cell}}} \mathrm{d}\boldsymbol{r} \sum_{A} \rho^A(\boldsymbol{r}) \int_{\mathbb{R}^3} \mathrm{d}\boldsymbol{r}' \frac{\sum_B \rho^B(\boldsymbol{r}')}{|\boldsymbol{r} - \boldsymbol{r}'|} - E_{\mathrm{self}} + E_{\mathrm{ovrl}}, \tag{S33}$$

where ρ^A represents a Gaussian charge distribution centered at the classical nucleus position:

$$\rho^A(\boldsymbol{r}) = -\frac{Z_A}{\left(R_A^{\mathrm{c}}\right)^3} \pi^{-3/2} \exp\left[-\left(\frac{\boldsymbol{r} - \boldsymbol{R}_A}{R_A^{\mathrm{c}}}\right)^2\right]. \tag{S34}$$

The self-energy correction term is:

$$E_{\mathrm{self}} = \sum_{A \in \text{unit cell}} \frac{1}{\sqrt{2\pi}} \frac{Z_A^2}{R_A^{\mathrm{c}}}, \tag{S35}$$

and the overlap correction term is:

$$\begin{aligned} E_{\mathrm{ovrl}} = {} & \frac{1}{2} \sum_{A,B \in \text{unit cell}, A \neq B} \frac{Z_A Z_B}{|\boldsymbol{R}_A - \boldsymbol{R}_B|} \operatorname{erfc}\left(\frac{|\boldsymbol{R}_A - \boldsymbol{R}_B|}{\sqrt{\left(R_A^{\mathrm{c}}\right)^2 + \left(R_B^{\mathrm{c}}\right)^2}}\right) \\ & + \frac{1}{2} \sum_{\boldsymbol{n} \neq (0,0,0)} \sum_{A \in \text{unit cell}} \sum_{B \in \text{unit cell}} \frac{Z_A Z_B}{|\boldsymbol{R}_A - \boldsymbol{R}_B - \boldsymbol{T}_{\boldsymbol{n}}|} \operatorname{erfc}\left(\frac{|\boldsymbol{R}_A - \boldsymbol{R}_B - \boldsymbol{T}_{\boldsymbol{n}}|}{\sqrt{\left(R_A^{\mathrm{c}}\right)^2 + \left(R_B^{\mathrm{c}}\right)^2}}\right). \end{aligned} \tag{S36}$$

The Gaussian-smeared ion generates the long-range part of the Coulomb potential:

$$\int_{\mathbb{R}^3} \mathrm{d}\boldsymbol{r}' \frac{\rho^A(\boldsymbol{r}')}{|\boldsymbol{r}-\boldsymbol{r}'|} = -\frac{Z_A}{|\boldsymbol{r}-\boldsymbol{R}_A|}\mathrm{erf}\left(\frac{|\boldsymbol{r}-\boldsymbol{R}_A|}{R_A^{\mathrm{c}}}\right), \tag{S37}$$

The total electrostatic energy per unit cell, including contributions from electrons, quantum nuclei, and classical nuclei, can be expressed as:

$$\begin{aligned}
&\int_{\Omega_{\mathrm{cell}}} \mathrm{d}\boldsymbol{r} V_{\mathrm{ext}}(\boldsymbol{r}) \left[\rho^{\mathrm{e}}(\boldsymbol{r}) - \sum_{a\in\text{unit cell}} Z_a \sum_{\boldsymbol{n}} \rho_a^{\mathrm{n}}(\boldsymbol{r}+\boldsymbol{T}_{\boldsymbol{n}})\right] \\
&\quad + E_{\mathrm{H}}\left[\rho^{\mathrm{e}}(\boldsymbol{r}) - \sum_{a\in\text{unit cell}} Z_a \sum_{\boldsymbol{n}} \rho_a^{\mathrm{n}}(\boldsymbol{r}+\boldsymbol{T}_{\boldsymbol{n}})\right] - \sum_{a\in\text{unit cell}} E_{\mathrm{H}}\left[Z_a\rho_a^{\mathrm{n}}\right] + E_{\mathrm{nuc}} \\
&= -\int_{\Omega_{\mathrm{cell}}} \mathrm{d}\boldsymbol{r} \left[\rho^{\mathrm{e}}(\boldsymbol{r}) - \sum_{a\in\text{unit cell}} Z_a \sum_{\boldsymbol{n}} \rho_a^{\mathrm{n}}(\boldsymbol{r}+\boldsymbol{T}_{\boldsymbol{n}})\right] \\
&\quad \times \sum_A \frac{Z_A}{|\boldsymbol{r}-\boldsymbol{R}_A|}\left[\mathrm{erf}\left(\frac{|\boldsymbol{r}-\boldsymbol{R}_A|}{R_A^{\mathrm{c}}}\right) + \mathrm{erfc}\left(\frac{|\boldsymbol{r}-\boldsymbol{R}_A|}{R_A^{\mathrm{c}}}\right)\right] \\
&\quad + \frac{1}{2}\int_{\Omega_{\mathrm{cell}}} \mathrm{d}\boldsymbol{r} \int_{\mathbb{R}^3} \mathrm{d}\boldsymbol{r}' \frac{1}{|\boldsymbol{r}-\boldsymbol{r}'|}\left[\rho^{\mathrm{e}}(\boldsymbol{r}) - \sum_{a\in\text{unit cell}} Z_a \sum_{\boldsymbol{n}} \rho_a^{\mathrm{n}}(\boldsymbol{r}+\boldsymbol{T}_{\boldsymbol{n}})\right] \\
&\quad \times \left[\rho^{\mathrm{e}}(\boldsymbol{r}') - \sum_{a\in\text{unit cell}} Z_a \sum_{\boldsymbol{n}} \rho_a^{\mathrm{n}}(\boldsymbol{r}'+\boldsymbol{T}_{\boldsymbol{n}})\right] \\
&\quad - \frac{1}{2}\sum_{a\in\text{unit cell}} Z_a^2 \int_{\mathbb{R}^3} \mathrm{d}\boldsymbol{r} \int_{\mathbb{R}^3} \mathrm{d}\boldsymbol{r}' \frac{\rho_a^{\mathrm{n}}(\boldsymbol{r})\rho_a^{\mathrm{n}}(\boldsymbol{r}')}{|\boldsymbol{r}-\boldsymbol{r}'|} \\
&\quad + \frac{1}{2}\int_{\Omega_{\mathrm{cell}}} \mathrm{d}\boldsymbol{r} \sum_A \rho^A(\boldsymbol{r}) \int_{\mathbb{R}^3} \mathrm{d}\boldsymbol{r}' \frac{\sum_B \rho^B(\boldsymbol{r}')}{|\boldsymbol{r}-\boldsymbol{r}'|} - E_{\mathrm{self}} + E_{\mathrm{ovrl}}
\end{aligned} \tag{S38}$$

$$\begin{aligned}
&= E_{\mathrm{H}}\left[\rho^{\mathrm{e}}(\boldsymbol{r}) - \sum_{a\in\text{unit cell}} Z_a \sum_{\boldsymbol{n}} \rho_a^{\mathrm{n}}(\boldsymbol{r}+\boldsymbol{T}_{\boldsymbol{n}}) + \sum_A \rho^A(\boldsymbol{r})\right] \\
&\quad - \frac{1}{2}\sum_{a\in\text{unit cell}} Z_a^2 \int_{\mathbb{R}^3} \mathrm{d}\boldsymbol{r} \int_{\mathbb{R}^3} \mathrm{d}\boldsymbol{r}' \frac{\rho_a^{\mathrm{n}}(\boldsymbol{r})\rho_a^{\mathrm{n}}(\boldsymbol{r}')}{|\boldsymbol{r}-\boldsymbol{r}'|} \\
&\quad - \int_{\Omega_{\mathrm{cell}}} \mathrm{d}\boldsymbol{r} \rho^{\mathrm{e}}(\boldsymbol{r}) \sum_A \frac{Z_A}{|\boldsymbol{r}-\boldsymbol{R}_A|}\mathrm{erfc}\left(\frac{|\boldsymbol{r}-\boldsymbol{R}_A|}{R_A^{\mathrm{c}}}\right) \\
&\quad + \sum_{a\in\text{unit cell}} Z_a \int_{\mathbb{R}^3} \mathrm{d}\boldsymbol{r} \rho_a^{\mathrm{n}}(\boldsymbol{r}) \sum_A \frac{Z_A}{|\boldsymbol{r}-\boldsymbol{R}_A|}\mathrm{erfc}\left(\frac{|\boldsymbol{r}-\boldsymbol{R}_A|}{R_A^{\mathrm{c}}}\right) - E_{\mathrm{self}} + E_{\mathrm{ovrl}}
\end{aligned} \tag{S39}$$

$$\equiv E_{\mathrm{H}}\left[\rho^{\mathrm{e}}(\boldsymbol{r}) - \sum_{a\in\text{unit cell}} Z_a \sum_{\boldsymbol{n}} \rho_a^{\mathrm{n}}(\boldsymbol{r}+\boldsymbol{T}_{\boldsymbol{n}}) + \sum_A \rho^A(\boldsymbol{r})\right] - \sum_{a\in\text{unit cell}} E_{\mathrm{H}}\left[Z_a\rho_a^{\mathrm{n}}\right]$$

$$+\int_{\Omega_{\text{cell}}} \mathrm{d}\boldsymbol{r}\rho^{\mathrm{e}}(\boldsymbol{r})V_{\text{ext}}^{\text{short}}(\boldsymbol{r}) - \sum_{a\in\text{unit cell}} Z_a \int_{\mathbb{R}^3} \mathrm{d}\boldsymbol{r}\rho_a^{\mathrm{n}}(\boldsymbol{r})V_{\text{ext}}^{\text{short}}(\boldsymbol{r}) - E_{\text{self}} + E_{\text{ovrl}}, \tag{S40}$$

where self-interactions of quantum nuclei are explicitly removed. The final expression allows us to compute the total electrostatic energy as the sum of: (1) the Hartree energy of the total charge density (which is neutral on average), (2) short-range external potential energies, and (3) correction terms. The short-range terms can be efficiently evaluated in real space due to the exponential decay of the complementary error function. It is worthy to mention that quantum nuclear self-interactions do not need to be explicitly evaluated, as we will show later.

While this approach effectively addresses the challenges of long-range Coulomb interactions from classical nuclei, we must still contend with the computational complexity of representing the total charge density, which contains both smooth (valence electron) and sharply varying (core electron and quantum nuclear) components. To handle this efficiently, we employ the Gaussian-augmented plane wave method described in the following section.

S2.3 Gaussian-augmented plane wave method with quantum nuclei

The Gaussian-augmented plane wave (GAPW) method provides an elegant solution to the challenge of efficiently representing densities that exhibit both smooth and sharply varying regions in space. This approach divides the system into atomic regions (spheres around each atom, denoted as U_A for atom A) and an interstitial region (I), allowing different computational treatments optimized for each domain.

The key insight of the GAPW method is the decomposition of the electron density into global soft, local hard, and local soft contributions:

$$\rho^{\mathrm{e}}(\boldsymbol{r}) = \tilde{\rho}^{\mathrm{e}}(\boldsymbol{r}) + \sum_A \rho_A^{\mathrm{e},1}(\boldsymbol{r}) - \tilde{\rho}_A^{\mathrm{e},1}(\boldsymbol{r}), \tag{S41}$$

where $\tilde{\rho}^{\mathrm{e}}$ represents the smooth global electronic density, $\rho_A^{\mathrm{e},1}$ corresponds to local "hard" densities centered around atom A, and $\tilde{\rho}_A^{\mathrm{e},1}$ denotes their corresponding soft counterparts.

This decomposition is constructed to satisfy specific spatial requirements:

- Within atomic regions: $\rho_A^{\mathrm{e},1}(\boldsymbol{r}) = \rho^{\mathrm{e}}(\boldsymbol{r})$ for $\boldsymbol{r} \in U_A$;
- In the interstitial region: $\tilde{\rho}^{\mathrm{e}}(\boldsymbol{r}) = \rho^{\mathrm{e}}(\boldsymbol{r})$ for $\boldsymbol{r} \in I$;
- The local soft density $\tilde{\rho}_A^{\mathrm{e},1}$ serves as the connecting piece by matching the local hard density outside atomic regions ($\tilde{\rho}_A^{\mathrm{e},1}(\boldsymbol{r}) = \rho_A^{\mathrm{e},1}(\boldsymbol{r})$ for $\boldsymbol{r} \notin U_A$) and matching the global soft density inside atomic regions ($\tilde{\rho}_A^{\mathrm{e},1}(\boldsymbol{r}) = \tilde{\rho}^{\mathrm{e}}(\boldsymbol{r})$ for $\boldsymbol{r} \in U_A$).

For quantum nuclei, we employ a similar decomposition:

$$\rho_a^{\mathrm{n}}(\boldsymbol{r}) = \rho_a^{\mathrm{n}}(\boldsymbol{r}) - \tilde{\rho}_a^{\mathrm{n}}(\boldsymbol{r}) + \tilde{\rho}_a^{\mathrm{n}}(\boldsymbol{r}), \tag{S42}$$

and the soft density also satisfies the requirement

$$\tilde{\rho}_a^{\mathrm{n}}(\boldsymbol{r}) = \rho_a^{\mathrm{n}}(\boldsymbol{r}), \quad \boldsymbol{r} \notin U_A. \tag{S43}$$

This separates the nuclear density into its sharply varying core component ($\rho_a^{\mathrm{n}} - \tilde{\rho}_a^{\mathrm{n}}$) confined within the atomic region, and its smooth tail ($\tilde{\rho}_a^{\mathrm{n}}$) extending into the interstitial region. In practical implementations, this decomposition is achieved by identifying the soft component of nuclear Gaussian basis functions according to their exponents and angular momenta, as well as the radius of the atomic region. Primitive Gaussian functions with values at sphere boundaries exceeding a threshold are considered "soft" functions that primarily contribute to the soft tail.

A crucial feature of the GAPW method is the introduction of compensation charges that reproduce the multipole moments of charge distributions within each atomic region. These compensation charges enable efficient separation of the Coulomb interaction into local and

global components. The compensation charge density is expressed as:

$$\rho^0(\boldsymbol{r}) = \sum_A \rho_A^0(\boldsymbol{r}), \tag{S44}$$

and each atomic component is localized in its atomic region. Because only the multipole moments of ρ_A^0 are of interest and its shape is not important, it is represented by a Gaussian-type radial basis function with real spherical harmonics as its angular part:

$$\rho_A^0(\boldsymbol{r}) = \sum_{l=0}^{\infty} \sum_{m=-l}^{l} Q_A^{lm} g_A^{lm}(\boldsymbol{r}). \tag{S45}$$

The exponent of the radial part of the Gaussian basis function g_A^{lm} is chosen such that ρ_A^0 has negligible values outside U_A, while the exponent is not too large either, such that ρ^0 can also be expressed by plane waves.

The multipole moments Q_A^{lm} account for the components of the total charge density that are strictly within the atomic regions. The expressions differ for classical and quantum nuclei. For atoms with classical nuclei:

$$Q_A^{lm} = q_{lm}\left[\rho_A^{\mathrm{e},1} - \tilde{\rho}_A^{\mathrm{e},1} + \rho^A\right]. \tag{S46}$$

For atoms with quantum nuclei, the multipole moments account for the quantum nuclear density contribution:

$$Q_a^{lm} = q_{lm}\left[\rho_a^{\mathrm{e},1} - \tilde{\rho}_a^{\mathrm{e},1} - Z_a\left(\rho_a^{\mathrm{n}} - \tilde{\rho}_a^{\mathrm{n}}\right)\right]. \tag{S47}$$

The definition of q_{lm} is

$$q_{lm}[\rho] \equiv \frac{4\pi}{2l+1} \int_0^{2\pi} \mathrm{d}\phi \int_0^{\pi} \mathrm{d}\theta \sin\theta \int_0^{\infty} \mathrm{d}r r^{l+2} \rho(r,\theta,\phi) S_l^m(\theta,\phi), \tag{S48}$$

with the origin of the spherical coordinate system chosen at the position of the corresponding atom center, and S_l^m denoting real spherical harmonics.

With this density decomposition and compensation charge framework established, the Hartree energy of the total charge density can be expressed as:

$$
\begin{aligned}
&E_{\mathrm{H}}\left[\rho^{\mathrm{e}}(\boldsymbol{r})-\sum_{a\in\text{unit cell}} Z_a\sum_{\boldsymbol{n}}\rho^{\mathrm{n}}_a(\boldsymbol{r}+\boldsymbol{T}_{\boldsymbol{n}})+\sum_{A}\rho^{A}(\boldsymbol{r})\right]\\
&=E_{\mathrm{H}}\left[\tilde{\rho}^{\mathrm{e}}(\boldsymbol{r})-\sum_{a\in\text{unit cell}} Z_a\sum_{\boldsymbol{n}}\tilde{\rho}^{\mathrm{n},1}_a(\boldsymbol{r}+\boldsymbol{T}_{\boldsymbol{n}})+\rho^{0}(\boldsymbol{r})\right.\\
&\quad+\sum_{a\in\text{unit cell}}\sum_{\boldsymbol{n}}\rho^{\mathrm{e},1}_a(\boldsymbol{r}+\boldsymbol{T}_{\boldsymbol{n}})-\tilde{\rho}^{\mathrm{e},1}_a(\boldsymbol{r}+\boldsymbol{T}_{\boldsymbol{n}})-Z_a\left(\rho^{\mathrm{n}}_a(\boldsymbol{r}+\boldsymbol{T}_{\boldsymbol{n}})-\tilde{\rho}^{\mathrm{n}}_a(\boldsymbol{r}+\boldsymbol{T}_{\boldsymbol{n}})\right)-\rho^{0}_a(\boldsymbol{r}+\boldsymbol{T}_{\boldsymbol{n}})\\
&\quad\left.+\sum_{A\in\text{classical}}\rho^{\mathrm{e},1}_A-\tilde{\rho}^{\mathrm{e},1}_A+\rho^{A}-\rho^{0}_A\right] \qquad \text{(S49)}\\
&=E_{\mathrm{H}}\left[\tilde{\rho}^{\mathrm{e}}(\boldsymbol{r})-\sum_{a\in\text{unit cell}} Z_a\sum_{\boldsymbol{n}}\tilde{\rho}^{\mathrm{n}}_a(\boldsymbol{r}+\boldsymbol{T}_{\boldsymbol{n}})+\rho^{0}(\boldsymbol{r})\right]\\
&\quad+\sum_{a\in\text{unit cell}}E_{\mathrm{H}}\left[\rho^{\mathrm{e},1}_a-Z_a\rho^{\mathrm{n}}_a\right]-E_{\mathrm{H}}\left[\tilde{\rho}^{\mathrm{e},1}_a-Z_a\tilde{\rho}^{\mathrm{n}}_a+\rho^{0}_a\right]\\
&\quad+\sum_{\substack{A\in\text{classical}\\ A\in\text{unit cell}}}E_{\mathrm{H}}\left[\rho^{\mathrm{e},1}_A+\rho^{A}\right]-E_{\mathrm{H}}\left[\tilde{\rho}^{\mathrm{e},1}_A+\rho^{0}_A\right]. \qquad \text{(S50)}
\end{aligned}
$$

This formulation provides significant computational efficiency by evaluating the smooth global density contribution in reciprocal space using fast Fourier transforms, while handling the local density contributions through direct numerical integration on local mesh grids.

It is worthy to note that, instead of explicitly calculating these terms, we employ a reformulation:

$$
E_{\mathrm{H}}\left[\rho^{\mathrm{e},1}_a-Z_a\rho^{\mathrm{n}}_a\right]-E_{\mathrm{H}}\left[Z_a\rho^{\mathrm{n}}_a\right]=E_{\mathrm{H}}\left[\rho^{\mathrm{e},1}_a\right]-Z_a\iint \mathrm{d}\boldsymbol{r}\,\mathrm{d}\boldsymbol{r}'\frac{\rho^{\mathrm{e},1}_a(\boldsymbol{r})\rho^{\mathrm{n}}_a(\boldsymbol{r}')}{|\boldsymbol{r}-\boldsymbol{r}'|}. \qquad \text{(S51)}
$$

This eliminates the need to explicitly calculate the nuclear self-interaction, requiring only the evaluation of the electron-nuclear attraction term.

S2.4 Total energy expression

Assuming a semi-local exchange-correlation functional is used, the exchange-correlation energy of electrons ($E^{\mathrm{e}}_{\mathrm{xc}}[\rho^{\mathrm{e}}]$) can have a decomposition that is similar to the Hartree energy:[S2]

$$E^{\mathrm{e}}_{\mathrm{xc}}[\rho^{\mathrm{e}}] = E^{\mathrm{e}}_{\mathrm{xc}}[\rho^{\mathrm{e}}|_I] + \sum_A E^{\mathrm{e}}_{\mathrm{xc}}[\rho^{\mathrm{e}}|_{U_A}] \tag{S52}$$

$$= E^{\mathrm{e}}_{\mathrm{xc}}[\tilde{\rho}^{\mathrm{e}}|_I] + \sum_A E^{\mathrm{e}}_{\mathrm{xc}}\left[\rho^{\mathrm{e},1}_A|_{U_A}\right] \tag{S53}$$

$$\begin{aligned}
= & E^{\mathrm{e}}_{\mathrm{xc}}[\tilde{\rho}^{\mathrm{e}}|_I] + \sum_A E^{\mathrm{e}}_{\mathrm{xc}}\left[\rho^{\mathrm{e},1}_A|_{U_A}\right] \\
& + \sum_A E^{\mathrm{e}}_{\mathrm{xc}}\left[\tilde{\rho}^{\mathrm{e},1}_A|_{U_A}\right] - E^{\mathrm{e}}_{\mathrm{xc}}\left[\tilde{\rho}^{\mathrm{e},1}_A|_{U_A}\right] \quad (=0) \\
& + E^{\mathrm{e}}_{\mathrm{xc}}\left[\sum_A \rho^{\mathrm{e},1}_A|_I\right] - E^{\mathrm{e}}_{\mathrm{xc}}\left[\sum_A \tilde{\rho}^{\mathrm{e},1}_A|_I\right] \quad (=0)
\end{aligned} \tag{S54}$$

$$\begin{aligned}
= & E^{\mathrm{e}}_{\mathrm{xc}}[\tilde{\rho}^{\mathrm{e}}|_I] + \sum_A E^{\mathrm{e}}_{\mathrm{xc}}\left[\tilde{\rho}^{\mathrm{e},1}_A|_{U_A}\right] \\
& + \sum_A E^{\mathrm{e}}_{\mathrm{xc}}\left[\rho^{\mathrm{e},1}_A|_{U_A}\right] + E^{\mathrm{e}}_{\mathrm{xc}}\left[\sum_A \rho^{\mathrm{e},1}_A|_I\right] \\
& - \sum_A E^{\mathrm{e}}_{\mathrm{xc}}\left[\tilde{\rho}^{\mathrm{e},1}_A|_{U_A}\right] - E^{\mathrm{e}}_{\mathrm{xc}}\left[\sum_A \tilde{\rho}^{\mathrm{e},1}_A|_I\right]
\end{aligned} \tag{S55}$$

$$= E^{\mathrm{e}}_{\mathrm{xc}}[\tilde{\rho}^{\mathrm{e}}] + E^{\mathrm{e}}_{\mathrm{xc}}\left[\sum_A \rho^{\mathrm{e},1}_A\right] - E^{\mathrm{e}}_{\mathrm{xc}}\left[\sum_A \tilde{\rho}^{\mathrm{e},1}_A\right] \tag{S56}$$

$$\approx E^{\mathrm{e}}_{\mathrm{xc}}[\tilde{\rho}^{\mathrm{e}}] + \sum_A E^{\mathrm{e}}_{\mathrm{xc}}\left[\rho^{\mathrm{e},1}_A\right] - \sum_A E^{\mathrm{e}}_{\mathrm{xc}}\left[\tilde{\rho}^{\mathrm{e},1}_A\right], \tag{S57}$$

assuming one-center densities decay reasonably fast when they go outside corresponding atomic regions, such that the one-body expansions are accurate enough.

We will omit the multicomponent correlation energy functional in the current development. The comprehensive total energy per unit cell thus becomes:

$$E\left[\rho^{\mathrm{e}}, \rho^{\mathrm{n}}_1, \rho^{\mathrm{n}}_2, \cdots, \rho^{\mathrm{n}}_{N^{\mathrm{n}}}\right] = T^{\mathrm{e}}_{\mathrm{s}}[\rho^{\mathrm{e}}] + \sum_{a=1}^{N^{\mathrm{n}}} T^{\mathrm{n}}_{\mathrm{s}}\left[\rho^{\mathrm{n}}_a\right] + \int_{\Omega_{\mathrm{cell}}} \mathrm{d}\boldsymbol{r}\,\rho^{\mathrm{e}}(\boldsymbol{r}) V^{\mathrm{short}}_{\mathrm{ext}}(\boldsymbol{r})$$

$$
\begin{aligned}
&- \sum_{a \in \text{unit cell}} Z_a \int_{\mathbb{R}^3} \mathrm{d}\boldsymbol{r} \rho_a^{\mathrm{n}}(\boldsymbol{r}) V_{\mathrm{ext}}^{\mathrm{short}}(\boldsymbol{r}) \\
&+ E_{\mathrm{H}} \left[\tilde{\rho}^{\mathrm{e}}(\boldsymbol{r}) - \sum_{a \in \text{unit cell}} Z_a \sum_{\boldsymbol{n}} \tilde{\rho}_a^{\mathrm{n}}(\boldsymbol{r} + \boldsymbol{T_n}) + \rho^0(\boldsymbol{r}) \right] \\
&+ \sum_{a \in \text{unit cell}} E_{\mathrm{H}} \left[\rho_a^{\mathrm{e},1} - Z_a \rho_a^{\mathrm{n}} \right] - E_{\mathrm{H}} \left[Z_a \rho_a^{\mathrm{n}} \right] - E_{\mathrm{H}} \left[\tilde{\rho}_a^{\mathrm{e},1} - Z_a \tilde{\rho}_a^{\mathrm{n}} + \rho_a^0 \right] \\
&+ \sum_{\substack{A \in \text{classical} \\ A \in \text{unit cell}}} E_{\mathrm{H}} \left[\rho_A^{\mathrm{e},1} + \rho^A \right] - E_{\mathrm{H}} \left[\tilde{\rho}_A^{\mathrm{e},1} + \rho_A^0 \right] - E_{\mathrm{self}} + E_{\mathrm{ovrl}} \\
&+ E_{\mathrm{xc}}^{\mathrm{e}}[\tilde{\rho}^{\mathrm{e}}] + \sum_{A \in \text{unit cell}} E_{\mathrm{xc}}^{\mathrm{e}} \left[\rho_A^{\mathrm{e},1} \right] - E_{\mathrm{xc}}^{\mathrm{e}} \left[\tilde{\rho}_A^{\mathrm{e},1} \right]. \qquad \text{(S58)}
\end{aligned}
$$

This comprehensive expression encompasses all relevant energy contributions for a periodic system with quantum nuclei, including kinetic energy, external potential interactions, Hartree energy, and electronic exchange-correlation energy. The distinguishable-particle periodic CNEO-DFT can in principle utilize existing electron-proton correlation (epc) functionals to construct the multicomponent correlation (see main text), and its implementation and investigation are left for future studies.

S2.5 Coupled nuclear and electronic equations

Since we have omitted the multicomponent correlation energy functional in our current implementation, the nuclear and electronic Kohn-Sham equations are coupled solely through Coulombic interactions.

For the electronic system, we denote contracted Gaussian basis functions as φ_μ^{e}, with $\tilde{\varphi}_\mu^{\mathrm{e}}$ representing the corresponding soft density basis (obtained by setting contraction coefficients for large-exponent Gaussians in φ_μ^{e} to zero). The functions $\chi_\mu^{\mathrm{e},A}$ and $\tilde{\chi}_\mu^{\mathrm{e},A}$ are contracted Gaussian basis functions for local densities centered at atom A, utilizing only primitive Gaussian functions centered at A. These are calculated using local projectors at A to enable Gaussian basis functions at A to represent density contributions from neighboring atoms. For the a-th quantum nucleus, we have $\varphi_\mu^{\mathrm{n},a}$ and $\tilde{\varphi}_\mu^{\mathrm{n},a}$ as the basis functions, typically in

uncontracted form.

The electronic and (unconstrained) nuclear Kohn-Sham Hamiltonian matrix elements can be derived from the total energy with respect to the density matrices. The electronic Hamiltonian takes the form:

$$\begin{aligned}
H^{\mathrm{e}}_{\mu\nu} &= \langle\varphi^{\mathrm{e}}_{\mu}\left|-\frac{\hbar^2}{2m_{\mathrm{e}}}\nabla^2 + V^{\mathrm{short}}_{\mathrm{ext}}\right|\varphi^{\mathrm{e}}_{\nu}\rangle \\
&+ \langle\tilde{\varphi}^{\mathrm{e}}_{\mu}\left|V_{\mathrm{H}}\left[\tilde{\rho}^{\mathrm{e}}(\boldsymbol{r}) - \sum_{a\in\text{unit cell}} Z_a \sum_{\boldsymbol{n}} \tilde{\rho}^{\mathrm{n}}_a(\boldsymbol{r}+\boldsymbol{T_n}) + \rho^0(\boldsymbol{r})\right] + V_{\mathrm{xc}}[\tilde{\rho}^{\mathrm{e}}]\right|\tilde{\varphi}^{\mathrm{e}}_{\nu}\rangle \\
&+ \sum_{\substack{a\in\text{quantum}\\ a\in\text{unit cell}}} \langle\chi^{\mathrm{e},a}_{\mu}\left|V_{\mathrm{H}}\left[\rho^{\mathrm{e},1}_a - Z_a\rho^{\mathrm{n}}_a\right] + V_{\mathrm{xc}}\left[\rho^{\mathrm{e},1}_a\right]\right|\chi^{\mathrm{e},a}_{\nu}\rangle \\
&- \langle\tilde{\chi}^{\mathrm{e},a}_{\mu}\left|V_{\mathrm{H}}\left[\tilde{\rho}^{\mathrm{e},1}_a - Z_a\tilde{\rho}^{\mathrm{n}}_a + \rho^0_a\right] + V_{\mathrm{xc}}\left[\tilde{\rho}^{\mathrm{e},1}_a\right]\right|\tilde{\chi}^{\mathrm{e},a}_{\nu}\rangle \\
&+ \sum_{\substack{A\in\text{classical}\\ A\in\text{unit cell}}} \langle\chi^{\mathrm{e},A}_{\mu}\left|V_{\mathrm{H}}\left[\rho^{\mathrm{e},1}_A + \rho^A\right] + V_{\mathrm{xc}}\left[\rho^{\mathrm{e},1}_A\right]\right|\chi^{\mathrm{e},A}_{\nu}\rangle \\
&- \langle\tilde{\chi}^{\mathrm{e},A}_{\mu}\left|V_{\mathrm{H}}\left[\tilde{\rho}^{\mathrm{e},1}_A + \rho^0_A\right] + V_{\mathrm{xc}}\left[\tilde{\rho}^{\mathrm{e},1}_A\right]\right|\tilde{\chi}^{\mathrm{e},A}_{\nu}\rangle \\
&+ \sum_{A\in\text{unit cell}} \sum_{lm} q_{lm}\left[\chi^{\mathrm{e},A}_{\mu}\chi^{\mathrm{e},A}_{\nu} - \tilde{\chi}^{\mathrm{e},A}_{\mu}\tilde{\chi}^{\mathrm{e},A}_{\nu}\right] \\
&\times \int_{\mathbb{R}^3} \mathrm{d}\boldsymbol{r} V_{\mathrm{H}}\left[\tilde{\rho}^{\mathrm{e}}(\boldsymbol{r}) - \sum_{a\in\text{unit cell}} Z_a \sum_{\boldsymbol{n}} \tilde{\rho}^{\mathrm{n}}_a(\boldsymbol{r}+\boldsymbol{T_n}) + \rho^0(\boldsymbol{r})\right](\boldsymbol{r}) g^{lm}_A(\boldsymbol{r}) \\
&- \sum_{\substack{a\in\text{quantum}\\ a\in\text{unit cell}}} \sum_{lm} q_{lm}\left[\chi^{\mathrm{e},a}_{\mu}\chi^{\mathrm{e},a}_{\nu} - \tilde{\chi}^{\mathrm{e},a}_{\mu}\tilde{\chi}^{\mathrm{e},a}_{\nu}\right] \int_{\mathbb{R}^3} \mathrm{d}\boldsymbol{r} V_{\mathrm{H}}\left[\tilde{\rho}^{\mathrm{e},1}_a - Z_a\tilde{\rho}^{\mathrm{n}}_a + \rho^0_a\right](\boldsymbol{r}) g^{lm}_a(\boldsymbol{r}) \\
&- \sum_{\substack{A\in\text{classical}\\ A\in\text{unit cell}}} \sum_{lm} q_{lm}\left[\chi^{\mathrm{e},A}_{\mu}\chi^{\mathrm{e},A}_{\nu} - \tilde{\chi}^{\mathrm{e},A}_{\mu}\tilde{\chi}^{\mathrm{e},A}_{\nu}\right] \int_{\mathbb{R}^3} \mathrm{d}\boldsymbol{r} V_{\mathrm{H}}\left[\tilde{\rho}^{\mathrm{e},1}_A + \rho^0_A\right](\boldsymbol{r}) g^{lm}_A(\boldsymbol{r}), \qquad \text{(S59)}
\end{aligned}$$

where $V_{\mathrm{H}}[\rho]$ denotes the Hartree potential generated by charge density ρ. In this electronic Hamiltonian, basis functions μ and ν extend beyond the reference unit cell (the 0-th cell). In practical numerical implementations, the evaluation of $H^{\mathrm{e}}_{\mu\nu}$ is truncated using an overlap integral threshold, meaning that only terms with significant enough overlap $S_{\mu\nu}$ are included in the Hamiltonian evaluation.

After constructing the electronic Hamiltonian beyond the reference unit cell, the $\boldsymbol{k}$-

dependent electronic Hamiltonian for basis functions centered strictly within the reference cell is constructed as:

$$H^{\mathrm{e}}_{\mu\nu}(\boldsymbol{k}) = N_{\mu\boldsymbol{k}} N_{\nu\boldsymbol{k}} \sum_{\boldsymbol{n}} H^{\mathrm{e}}_{\mu\nu}(\boldsymbol{n}) \exp(\mathrm{i}\boldsymbol{k}\cdot\boldsymbol{T}_{\boldsymbol{n}}), \tag{S60}$$

where

$$H^{\mathrm{e}}_{\mu\nu}(\boldsymbol{n}) = H^{\mathrm{e}}_{\mu\nu'}, \tag{S61}$$

and basis function ν' is the image of basis function ν (translated from the reference cell to the $\boldsymbol{n}$-th cell via lattice vector $\boldsymbol{T}_{\boldsymbol{n}}$):

$$\varphi^{\mathrm{e}}_{\nu'}(\boldsymbol{r} - \boldsymbol{T}_{\boldsymbol{n}}) = \varphi^{\mathrm{e}}_{\nu}(\boldsymbol{r}). \tag{S62}$$

$N_{\mu\boldsymbol{k}}$ is the normalization factor that ensures proper normalization of the crystalline orbitals. This approach represents the standard crystalline orbital implementation when localized atomic orbitals serve as basis functions for a periodic system.[S3]

For the nuclear Hamiltonian of the a-th quantum nucleus, we have:

$$\begin{aligned}
H^{\mathrm{n},a}_{\mu\nu} = {} & \langle\varphi^{\mathrm{n},a}_{\mu}\left| -\frac{\hbar^2}{2m_{\mathrm{n}}}\nabla^2 - Z_a V^{\mathrm{short}}_{\mathrm{ext}} \right|\varphi^{\mathrm{n},a}_{\nu}\rangle \\
& - Z_a \langle\tilde{\varphi}^{\mathrm{n},a}_{\mu}\left| V_{\mathrm{H}}\left[\tilde{\rho}^{\mathrm{e}}(\boldsymbol{r}) - \sum_{b\in\text{unit cell}} Z_b \sum_{\boldsymbol{n}} \tilde{\rho}^{\mathrm{n}}_b(\boldsymbol{r}+\boldsymbol{T}_{\boldsymbol{n}}) + \rho^0(\boldsymbol{r})\right]\right|\tilde{\varphi}^{\mathrm{n},a}_{\nu}\rangle \\
& - Z_a \langle\varphi^{\mathrm{n},a}_{\mu}\left| V_{\mathrm{H}}\left[\rho^{\mathrm{e},1}_a\right]\right|\varphi^{\mathrm{n},a}_{\nu}\rangle + Z_a \langle\tilde{\varphi}^{\mathrm{n},a}_{\mu}\left| V_{\mathrm{H}}\left[\tilde{\rho}^{\mathrm{e},1}_a - Z_a\tilde{\rho}^{\mathrm{n}}_a + \rho^0_a\right]\right|\tilde{\varphi}^{\mathrm{n},a}_{\nu}\rangle \\
& - Z_a \sum_{lm} q_{lm}\left[\varphi^{\mathrm{n},a}_{\mu}\varphi^{\mathrm{n},a}_{\nu} - \tilde{\varphi}^{\mathrm{n},a}_{\mu}\tilde{\varphi}^{\mathrm{n},a}_{\nu}\right] \\
& \times \int_{\mathbb{R}^3} \mathrm{d}\boldsymbol{r} V_{\mathrm{H}}\left[\tilde{\rho}^{\mathrm{e}}(\boldsymbol{r}) - \sum_{b\in\text{unit cell}} Z_b \sum_{\boldsymbol{n}} \tilde{\rho}^{\mathrm{n}}_b(\boldsymbol{r}+\boldsymbol{T}_{\boldsymbol{n}}) + \rho^0(\boldsymbol{r})\right](\boldsymbol{r}) g^{lm}_a(\boldsymbol{r}) \\
& + Z_a \sum_{lm} q_{lm}\left[\varphi^{\mathrm{n},a}_{\mu}\varphi^{\mathrm{n},a}_{\nu} - \tilde{\varphi}^{\mathrm{n},a}_{\mu}\tilde{\varphi}^{\mathrm{n},a}_{\nu}\right] \int_{\mathbb{R}^3} \mathrm{d}\boldsymbol{r} V_{\mathrm{H}}\left[\tilde{\rho}^{\mathrm{e},1}_a - Z_a\tilde{\rho}^{\mathrm{n}}_a + \rho^0_a\right](\boldsymbol{r}) g^{lm}_a(\boldsymbol{r}).
\end{aligned} \tag{S63}$$

Note that in the nuclear Hamiltonian, the Hartree potential generated by local hard densities

includes only the Coulombic attraction from electrons, thus automatically removing nuclear self-Coulomb interactions. Unlike the electronic case, the nuclear problem is a localized single-particle problem, so the basis functions μ and ν in the nuclear Hamiltonian are strictly those centered at position $\boldsymbol{R}_a$ of the quantum nucleus.

S2.6 SCF procedure

While a simultaneous direct inversion in the iterative subspace (DIIS) approach for electrons and nuclei is preferable for molecular systems (as implemented in our fork of PySCF that has CNEO functionalities[S4]), we adopt a simpler approach in our periodic implementation to maintain compatibility with CP2K's various electronic SCF algorithms. It is still simultaneous, but the acceleration is only applied to the electronic subsystem.

The computational cost increase due to nuclear steps in each iteration is negligible because no new expensive operations are introduced—all nuclear-specific terms utilize the existing GAPW computational infrastructure. For nuclear Kohn-Sham equations, even hundreds of quantum nuclei contribute minimally to the overall computational time, as each nuclear eigenvalue problem involves only a small number of basis functions (e.g., 23 functions for PB4-D with spherical harmonics). This demonstrates a key computational advantage of the distinguishable-particle approximation: it reduces the nuclear problem to many independent small-scale eigenvalue problems rather than a single large-scale system. However, we do see slight increases in the number of electronic cycles as compared to conventional DFT calculations due to the simple simultaneous solution of coupled Kohn-Sham equations without a properly-designed acceleration technique that combines errors from all subsystems. In the future one might be able to integrate the nuclear density matrix update within the orbital transformation (OT) method, similar to the simultaneous multicomponent DIIS, to create a simultaneous OT for multicomponent calculations, such that a more efficient SCF can be achieved.

Our SCF procedure for periodic CNEO-DFT calculations follows the general workflow

illustrated below:

1. **Initialization**: The electronic system is solved using default smeared ion densities at all nuclear positions. Global and local Hartree potentials are computed at this stage. Nuclear soft densities and their potentials on local grids are initially set to zero. Since nuclear self-interaction is explicitly removed and no multicomponent correlation functional is currently implemented, we do not need an initial guess for the nuclear density matrix.

2. **Nuclear Kohn-Sham Iteration**: After each electronic iteration, all nuclear Kohn-Sham Hamiltonian matrices are constructed in parallel. We deliberately avoid pursuing full self-consistency of quantum nuclei between electronic cycles. This design choice is motivated by computational efficiency—nuclear micro-iterations would require frequent updates to nuclear soft densities and compensation charges (ρ^0), leading to multiple global and local Hartree potential recalculations that would significantly increase computational cost without necessarily improving overall convergence. Instead, we aim for simultaneous convergence of electrons and quantum nuclei. For unconstrained NEO-DFT, a single diagonalization without Hartree potential updates would suffice. However, CNEO-DFT requires iterative optimization of the Lagrange multiplier $\boldsymbol{f}_a$, necessitating multiple diagonalizations. We achieve this efficiently by temporarily fixing the underlying unconstrained NEO-DFT Kohn-Sham Hamiltonian in this step, and updating only $\boldsymbol{f}_a$. The updated nuclear densities that satisfy the position constraints are computed, along with the corresponding local Hartree potentials.

3. **Electronic Kohn-Sham Iteration**: With updated nuclear densities and local Hartree potentials, we proceed to the next electronic Kohn-Sham iteration. The compensation charges ρ^0 are updated based on the new electronic density and updated nuclear densities. Necessary Hartree potentials are computed using all updated densities. The electronic Kohn-Sham iteration may utilize the history of electronic orbitals and em-

ploy various acceleration techniques such as DIIS, OT, and density mixing to update the electron density more effectively. The electronic step is performed for only one Kohn-Sham iteration instead of seeking for immediate convergence, as guided by the principle of simultaneous convergence of the multicomponent system. Upon completion of the electronic iteration, the procedure returns to step 2.

4. **Convergence Criterion**: The SCF procedure is considered converged when the convergence criterion for the electronic system is satisfied. We do not explicitly monitor the nuclear density matrix difference between consecutive iterations because electronic convergence already implies minimal changes in the potential generated by quantum nuclei. Furthermore, the CNEO-DFT total energy typically converges sufficiently when the electronic criterion is met.

This simultaneous approach is the key to high computational efficiency (comparable to conventional DFT calculations). By focusing on simultaneous convergence of the coupled electron-nuclear system rather than pursuing separate self-consistency for the subsystems, we achieve practical calculations for periodic systems with quantum mechanical treatment of selected nuclei.

S2.7 Analytic gradients

Analytic gradients of the total energy with respect to nuclear coordinates are essential for geometry optimization and molecular dynamics simulations. In the CNEO-DFT framework, we must consider two types of coordinate derivatives:

1. **Gradients with respect to classical nuclear coordinates**: The coordinate change affects the classical smeared ion density ρ^A, the short-range external potential $V_{\text{ext}}^{\text{short}}$, electronic basis functions φ^{e} centered at the perturbed atom, the projectors used to calculate χ^{e} for the perturbed atom, and the Gaussian basis functions g_A for the compensating charge at the perturbed atom.

2. **Gradients with respect to quantum nuclear coordinates**: The quantum nuclear position represents the expectation position of the ground state nuclear orbital, which serves as the electronic/nuclear basis center. CNEO-DFT effectively constrains the nuclear orbital expectation position to match the basis center. Coordinate changes affect electronic basis functions φ^{e} and nuclear basis functions φ^{n} centered at the perturbed atom, the projectors used to calculate χ^{e} for the perturbed atom, and the Gaussian basis functions for the compensating charge at the perturbed atom.

Most of the analytic gradient terms are already implemented in CP2K as part of the standard GAPW force evaluation.[S5,S6] For CNEO-DFT, we need to evaluate additional terms associated with quantum nuclei, both for classical coordinate changes and quantum expectation coordinate changes.

From the total energy expression, the terms unique to CNEO-DFT include:

1. Nuclear kinetic energy;
2. Nuclear (short-range) external potential energy;
3. Global Hartree energy contributions from nuclear soft densities;
4. Local Hartree terms for quantum nuclei due to nuclear hard and soft densities;
5. Global and local Hartree terms dependent on ρ^0, which implicitly includes quantum nuclear densities.

Due to the distinguishable-quantum-nuclei approximation, each nucleus has its own basis functions centered at the nuclear expectation position. Consequently, the nuclear overlap and kinetic matrices remain unchanged upon basis center modification, resulting in zero gradient contributions for these terms. We can conclude for entry 1, the gradient is zero. Zero nuclear overlap matrix gradients also lead to zero nuclear density matrix change gradients. This is because this gradient contribution is formulated as the product of the energy-weighted density matrix and overlap matrix gradients.

For remaining gradients with respect to basis functions, first let us investigate entry 4, the local Hartree energy term associated with the a-th quantum nucleus and its surrounding electrons:

$$\begin{aligned}
&E_{\mathrm{H}}\left[\rho_a^{\mathrm{e},1} - Z_a\rho_a^{\mathrm{n}}\right] - E_{\mathrm{H}}\left[Z_a\rho_a^{\mathrm{n}}\right] - E_{\mathrm{H}}\left[\tilde{\rho}_a^{\mathrm{e},1} - Z_a\tilde{\rho}_a^{\mathrm{n}} + \rho_a^0\right] \\
&= \frac{1}{2}\iint \mathrm{d}\boldsymbol{r}\,\mathrm{d}\boldsymbol{r}'\frac{\rho_a^{\mathrm{e},1}(\boldsymbol{r})\rho_a^{\mathrm{e},1}(\boldsymbol{r}')}{|\boldsymbol{r}-\boldsymbol{r}'|} - Z_a\iint \mathrm{d}\boldsymbol{r}\,\mathrm{d}\boldsymbol{r}'\frac{\rho_a^{\mathrm{e},1}(\boldsymbol{r})\rho_a^{\mathrm{n}}(\boldsymbol{r}')}{|\boldsymbol{r}-\boldsymbol{r}'|} \\
&\quad - \frac{1}{2}\iint \mathrm{d}\boldsymbol{r}\,\mathrm{d}\boldsymbol{r}'\frac{\left[\tilde{\rho}_a^{\mathrm{e},1}(\boldsymbol{r}) - Z_a\tilde{\rho}_a^{\mathrm{n}}(\boldsymbol{r}) + \rho_a^0(\boldsymbol{r})\right]\left[\tilde{\rho}_a^{\mathrm{e},1}(\boldsymbol{r}') - Z_a\tilde{\rho}_a^{\mathrm{n}}(\boldsymbol{r}') + \rho_a^0(\boldsymbol{r}')\right]}{|\boldsymbol{r}-\boldsymbol{r}'|},
\end{aligned} \tag{S64}$$

where all local densities are expanded by Gaussian basis functions at the same center $\boldsymbol{R}_a$:

$$\rho_a^{\mathrm{e},1}(\boldsymbol{r}-\boldsymbol{R}_a) = \sum_{\mu\nu\in\text{all e basis}} P_{\mu\nu}^{\mathrm{e}}\chi_\mu^{\mathrm{e},a}(\boldsymbol{r}-\boldsymbol{R}_a)\chi_\nu^{\mathrm{e},a}(\boldsymbol{r}-\boldsymbol{R}_a), \tag{S65}$$

$$\tilde{\rho}_a^{\mathrm{e},1}(\boldsymbol{r}-\boldsymbol{R}_a) = \sum_{\mu\nu\in\text{all e basis}} P_{\mu\nu}^{\mathrm{e}}\tilde{\chi}_\mu^{\mathrm{e},a}(\boldsymbol{r}-\boldsymbol{R}_a)\tilde{\chi}_\nu^{\mathrm{e},a}(\boldsymbol{r}-\boldsymbol{R}_a), \tag{S66}$$

$$\rho_a^{\mathrm{n}}(\boldsymbol{r}-\boldsymbol{R}_a) = \sum_{\mu\nu\in a} P_{\mu\nu}^{\mathrm{n},a}\varphi_\mu^{\mathrm{n},a}(\boldsymbol{r}-\boldsymbol{R}_a)\varphi_\nu^{\mathrm{n},a}(\boldsymbol{r}-\boldsymbol{R}_a), \tag{S67}$$

$$\tilde{\rho}_a^{\mathrm{n}}(\boldsymbol{r}-\boldsymbol{R}_a) = \sum_{\mu\nu\in a} P_{\mu\nu}^{\mathrm{n},a}\tilde{\varphi}_\mu^{\mathrm{n},a}(\boldsymbol{r}-\boldsymbol{R}_a)\tilde{\varphi}_\nu^{\mathrm{n},a}(\boldsymbol{r}-\boldsymbol{R}_a), \tag{S68}$$

and

$$\rho_a^0(\boldsymbol{r}-\boldsymbol{R}_a) = \sum_{l=0}^{\infty}\sum_{m=-l}^{l} q_{lm}\left[\rho_a^{\mathrm{e},1} - \tilde{\rho}_a^{\mathrm{e},1} - Z_a\left(\rho_a^{\mathrm{n}} - \tilde{\rho}_a^{\mathrm{n}}\right)\right] g_a^{lm}(\boldsymbol{r}-\boldsymbol{R}_a). \tag{S69}$$

Because the integral of products of Gaussian functions with the same center is independent of the center position, the gradient of the local Hartree term has no explicit dependence on the basis function center. The gradient contributions arise only through other components: $\chi_\mu^{\mathrm{e},a}$ and multipole moments.

The gradient of $\chi_\mu^{\mathrm{e},a}$ contributes due to changes in contraction coefficients from local projector calculations, but this term is already handled by the standard GAPW gradients. Similarly, the gradient contributions from multipole moments due to changes in $\chi_\mu^{\mathrm{e},a}$ are

included in the original GAPW gradient. The remaining nuclear density component involves:

$$q_{lm}\left[-Z_a\left(\rho_a^{\mathrm{n}}-\tilde{\rho}_a^{\mathrm{n}}\right)\right]=-Z_a\frac{4\pi}{2l+1}\int_0^{2\pi}\mathrm{d}\phi\int_0^{\pi}\mathrm{d}\theta\sin\theta\int_0^{\infty}\mathrm{d}r r^{l+2}S_l^m(\theta,\phi)$$
$$\times\sum_{\mu\nu\in a}P_{\mu\nu}^{\mathrm{n},a}\left[\varphi_\mu^{\mathrm{n},a}(\boldsymbol{r}-\boldsymbol{R}_a)\varphi_\nu^{\mathrm{n},a}(\boldsymbol{r}-\boldsymbol{R}_a)-\tilde{\varphi}_\mu^{\mathrm{n},a}(\boldsymbol{r}-\boldsymbol{R}_a)\tilde{\varphi}_\nu^{\mathrm{n},a}(\boldsymbol{r}-\boldsymbol{R}_a)\right]. \tag{S70}$$

Since the multipole moment is evaluated using $\boldsymbol{R}_a$ as the origin, the multipole moment no longer has explicit dependence on $\boldsymbol{R}_a$. Additionally, because quantum nuclei do not use χ but directly use the fixed φ basis functions for local densities, there is no gradient associated with local projector changes. Consequently, its gradient depends only on the density matrix $P_{\mu\nu}^{\mathrm{n},a}$:

$$\frac{\partial}{\partial\xi}q_{lm}\left[-Z_a\left(\rho_a^{\mathrm{n}}-\tilde{\rho}_a^{\mathrm{n}}\right)\right]=-Z_a\sum_{\mu\nu\in a}q_{lm}\left[\varphi_\mu^{\mathrm{n},a}\varphi_\nu^{\mathrm{n},a}-\tilde{\varphi}_\mu^{\mathrm{n},a}\tilde{\varphi}_\nu^{\mathrm{n},a}\right]\frac{\partial P_{\mu\nu}^{\mathrm{n},a}}{\partial\xi}, \tag{S71}$$

where ξ is a component of $\boldsymbol{R}_a$. This term, as it stands alone, is indeed non-zero. However, we do not separately evaluate this term. For the CNEO-DFT total energy gradient, contributions associated with nuclear density matrix changes are collected together, and in total they will produce zero gradients due to the zero nuclear overlap matrix gradients as mentioned before. This also implies we do not need to worry about entry 5.

For entry 3, the global Hartree energy contribution involving soft nuclear densities, the gradient is:

$$\begin{aligned}
&\frac{\partial}{\partial\xi}E_{\mathrm{H}}\left[\tilde{\rho}^{\mathrm{e}}(\boldsymbol{r})-\sum_{a\in\text{unit cell}}Z_a\sum_{\boldsymbol{n}}\tilde{\rho}_a^{\mathrm{n}}(\boldsymbol{r}+\boldsymbol{T}_{\boldsymbol{n}})+\rho^0(\boldsymbol{r})\right]\\
&=\int_{\Omega_{\text{cell}}}\mathrm{d}\boldsymbol{r}V_{\mathrm{H}}\left[\tilde{\rho}^{\mathrm{e}}(\boldsymbol{r})-\sum_{a\in\text{unit cell}}Z_a\sum_{\boldsymbol{n}}\tilde{\rho}_a^{\mathrm{n}}(\boldsymbol{r}+\boldsymbol{T}_{\boldsymbol{n}})+\rho^0(\boldsymbol{r})\right](\boldsymbol{r})\\
&\quad\times\frac{\partial}{\partial\xi}\left[\tilde{\rho}^{\mathrm{e}}(\boldsymbol{r})-\sum_{a\in\text{unit cell}}Z_a\sum_{\boldsymbol{n}}\tilde{\rho}_a^{\mathrm{n}}(\boldsymbol{r}+\boldsymbol{T}_{\boldsymbol{n}})+\rho^0(\boldsymbol{r})\right] \qquad\qquad (\text{S72})\\
&=\int_{\Omega_{\text{cell}}}\mathrm{d}\boldsymbol{r}V_{\mathrm{H}}\left[\tilde{\rho}^{\mathrm{e}}(\boldsymbol{r})-\sum_{a\in\text{unit cell}}Z_a\sum_{\boldsymbol{n}}\tilde{\rho}_a^{\mathrm{n}}(\boldsymbol{r}+\boldsymbol{T}_{\boldsymbol{n}})+\rho^0(\boldsymbol{r})\right](\boldsymbol{r})\frac{\partial}{\partial\xi}[\tilde{\rho}^{\mathrm{e}}(\boldsymbol{r})+\rho^0(\boldsymbol{r})]
\end{aligned}$$

$$- Z_a \sum_{a\in\text{unit cell}} \int_{\mathbb{R}^3} \mathrm{d}\boldsymbol{r} V_{\mathrm{H}} \left[\tilde{\rho}^{\mathrm{e}}(\boldsymbol{r}) - \sum_{b\in\text{unit cell}} Z_b \sum_{\boldsymbol{n}} \tilde{\rho}^{\mathrm{n}}_b(\boldsymbol{r}+\boldsymbol{T}_{\boldsymbol{n}}) + \rho^0(\boldsymbol{r})\right](\boldsymbol{r}) \frac{\partial}{\partial \xi} \tilde{\rho}^{\mathrm{n}}_a(\boldsymbol{r}). \quad \text{(S73)}$$

As mentioned before, the gradients of ρ^0 arising from multipole moments contain only nuclear density matrix contributions but no contribution from nuclear basis center changes. Therefore, the gradient of $\tilde{\rho}^{\mathrm{e}} + \rho^0$ due to electronic basis (φ^{e}, $\tilde{\varphi}^{\mathrm{e}}$, χ^{e} and $\tilde{\chi}^{\mathrm{e}}$) changes and ρ^0 basis (g_A^{lm}) changes are already included in the standard GAPW gradient. The remaining contribution comes from the explicit change of nuclear soft basis functions ($\tilde{\varphi}^{\mathrm{n}}$).

In summary, for CNEO-DFT, we need to evaluate gradients arising from nuclear basis changes from two sources (entries 2 and 3):

1. For classical coordinate changes: The gradient of the short-range external potential one-body integral of the quantum nucleus with respect to potential changes.
2. For quantum coordinate changes: The short-range external potential integral gradient with respect to basis center changes, and the integral of the global Hartree potential with the gradient of soft nuclear basis due to basis center changes.

The final expression for these nuclear-specific gradient contributions is:

$$\begin{aligned} g_{\xi,\mathrm{nuc}} = &- \sum_{a\in\text{unit cell}} Z_a \sum_{\mu\nu\in a} P^{\mathrm{n},a}_{\mu\nu} \left\{ \int_{\mathbb{R}^3} \mathrm{d}\boldsymbol{r} \frac{\partial}{\partial \xi} \left[\varphi^{\mathrm{n},a}_{\mu}(\boldsymbol{r}) \varphi^{\mathrm{n},a}_{\nu}(\boldsymbol{r}) V^{\text{short}}_{\text{ext}}(\boldsymbol{r})\right] \right. \\ &\left. + \int_{\mathbb{R}^3} \mathrm{d}\boldsymbol{r} V_{\mathrm{H}} \left[\tilde{\rho}^{\mathrm{e}}(\boldsymbol{r}) - \sum_{b\in\text{unit cell}} Z_b \sum_{\boldsymbol{n}} \tilde{\rho}^{\mathrm{n}}_b(\boldsymbol{r}+\boldsymbol{T}_{\boldsymbol{n}}) + \rho^0(\boldsymbol{r})\right](\boldsymbol{r}) \frac{\partial}{\partial \xi} \left[\tilde{\varphi}^{\mathrm{n},a}_{\mu}(\boldsymbol{r}) \tilde{\varphi}^{\mathrm{n},a}_{\nu}(\boldsymbol{r})\right] \right\}. \end{aligned} \quad \text{(S74)}$$

With these analytic gradient expressions implemented, CNEO-DFT with periodic boundary conditions enables efficient geometry optimization and molecular dynamics simulations that inherently account for nuclear quantum effects in extended systems.

S3 Numerical Methods for Quantum Reference Calculations

S3.1 Numerical solution to 2D nuclear Schrödinger equation with a periodic potential

To provide a quantum mechanical reference for our system, we directly solve the Schrödinger equation for a hydrogen nucleus moving in the two-dimensional periodic potential energy surface of the Pt(111) substrate. This section details the numerical approach used to obtain the quantum energy levels and wave functions that serve as a benchmark for our thermodynamic calculations.

We employ a plane wave expansion method combined with Bloch's theorem to efficiently handle the periodic boundary conditions. The potential energy surface is sampled on a real-space grid and transformed to reciprocal space using fast Fourier transforms (FFT).

For hydrogen adsorbed on the Pt(111) surface, we represent the two-dimensional periodic system with lattice vectors $\boldsymbol{a}_1$ and $\boldsymbol{a}_2$, and their corresponding reciprocal lattice vectors $\boldsymbol{b}_1$ and $\boldsymbol{b}_2$. The potential energy surface exhibits the periodicity:

$$V(\boldsymbol{r} + n_1\boldsymbol{a}_1 + n_2\boldsymbol{a}_2) = V(\boldsymbol{r}), \quad \forall n_1, n_2 \in \mathbb{Z}. \tag{S75}$$

This allows us to express the potential as a Fourier series:

$$V(\boldsymbol{r}) = \sum_{m_1\in\mathbb{Z}}\sum_{m_2\in\mathbb{Z}} \tilde{V}(m_1\boldsymbol{b}_1 + m_2\boldsymbol{b}_2)\exp[\mathrm{i}(m_1\boldsymbol{b}_1 + m_2\boldsymbol{b}_2)\cdot\boldsymbol{r}]. \tag{S76}$$

For notation convenience, we denote $\boldsymbol{m} = (m_1, m_2)$ and $\boldsymbol{G_m} = m_1\boldsymbol{b}_1 + m_2\boldsymbol{b}_2$. The Fourier components are calculated through:

$$\tilde{V}(\boldsymbol{G_m}) = \frac{1}{|\boldsymbol{a}_1 \times \boldsymbol{a}_2|}\int_{|\boldsymbol{a}_1\times\boldsymbol{a}_2|} \mathrm{d}\boldsymbol{r} V(\boldsymbol{r})\exp(-\mathrm{i}\boldsymbol{G_m}\cdot\boldsymbol{r}). \tag{S77}$$

These integrals are efficiently evaluated using fast Fourier transforms of the real-space grid representation of the potential.

According to Bloch's theorem, the wave functions for a particle in a periodic potential take the form:

$$\psi_{i\boldsymbol{k}}(\boldsymbol{r}) = \frac{1}{\sqrt{|\boldsymbol{a}_1 \times \boldsymbol{a}_2|}} \mathrm{e}^{\mathrm{i}\boldsymbol{k}\cdot\boldsymbol{r}} \sum_{\boldsymbol{m}} C_{i\boldsymbol{m}}(\boldsymbol{k}) \exp(\mathrm{i}\boldsymbol{G}_{\boldsymbol{m}} \cdot \boldsymbol{r}), \tag{S78}$$

where $C_{i\boldsymbol{m}}(\boldsymbol{k})$ are expansion coefficients that must be determined by solving the eigenvalue equation:

$$\sum_{\boldsymbol{m}'} H_{\boldsymbol{m}\boldsymbol{m}'}(\boldsymbol{k}) C_{i\boldsymbol{m}'}(\boldsymbol{k}) = E_{i\boldsymbol{k}} C_{i\boldsymbol{m}}(\boldsymbol{k}). \tag{S79}$$

The Hamiltonian matrix elements are given by:

$$H_{\boldsymbol{m}\boldsymbol{m}'}(\boldsymbol{k}) = \frac{\hbar^2}{2M}(\boldsymbol{k} + \boldsymbol{G}_{\boldsymbol{m}})^2 \delta_{\boldsymbol{m}\boldsymbol{m}'} + \tilde{V}(\boldsymbol{G}_{\boldsymbol{m}} - \boldsymbol{G}_{\boldsymbol{m}'}), \tag{S80}$$

where M is the mass of the hydrogen atom. The first term represents the kinetic energy, while the second term accounts for the potential energy in reciprocal space.

To obtain a complete quantum description, we sample the wave vector $\boldsymbol{k}$ within the first Brillouin zone:

$$\boldsymbol{k} = x_1 \boldsymbol{b}_1 + x_2 \boldsymbol{b}_2, \quad \forall x_1, x_2 \in \left[-\frac{1}{2}, \frac{1}{2}\right). \tag{S81}$$

In practical implementations, the resolution of the real-space grid for the PES imposes a cutoff on the maximum momentum in reciprocal space. The wave function expansion is typically truncated at half the momentum cutoff of the potential, which means the real-space grid for the wave function can be half as dense as the grid for the potential in each direction.

S3.2 Quantum statistical mechanics for thermodynamic properties

Once the quantum energy levels $E_{i\boldsymbol{k}}$ are obtained from the Schrödinger equation, we can calculate thermodynamic properties using quantum statistical mechanics. These calculations provide reference values for comparing different computational approaches.

The single-particle canonical ensemble partition function is given by:

$$q = \sum_i \sum_{\boldsymbol{k}} \exp\left(-\frac{E_{i\boldsymbol{k}}}{k_\mathrm{B}T}\right), \tag{S82}$$

where the summation over $\boldsymbol{k}$ spans the sampled points in the first Brillouin zone. For numerical calculations, we use a uniform $\boldsymbol{k}$-point mesh with a total of M points. This discretization corresponds to a Born-von Karman periodic boundary condition encompassing M unit cells.

The average partition function per unit cell is:

$$q_\mathrm{avg} \equiv \frac{q}{M} = \frac{1}{M} \sum_i \sum_{\boldsymbol{k}} \exp\left(-\frac{E_{i\boldsymbol{k}}}{k_\mathrm{B}T}\right). \tag{S83}$$

For a system with N hydrogen atoms, the total partition function is:

$$Q = \frac{q^N}{N!} = \frac{M^N q_\mathrm{avg}^N}{N!}. \tag{S84}$$

The coverage is defined as the ratio of particles to available sites:

$$\theta = \frac{N}{M}. \tag{S85}$$

From these definitions, we can derive the Helmholtz free energy per particle:

$$\frac{A}{N} = -\frac{1}{N} k_\mathrm{B}T \ln Q \tag{S86}$$

$$= -\frac{1}{N} k_{\mathrm{B}} T [N \ln q - N \ln N + N] \tag{S87}$$

$$= -k_{\mathrm{B}} T [\ln(q/N) + 1] \tag{S88}$$

$$= -k_{\mathrm{B}} T \left[\ln\left(\frac{q_{\mathrm{avg}}}{\theta} \right) + 1 \right]. \tag{S89}$$

The entropy per particle is obtained by differentiating the free energy with respect to temperature:

$$\frac{S}{N} = -\frac{\partial}{\partial T} \frac{A}{N} \tag{S90}$$

$$= k_{\mathrm{B}} \left[\ln\left(\frac{q_{\mathrm{avg}}}{\theta} \right) + 1 \right] + k_{\mathrm{B}} T \frac{1}{q_{\mathrm{avg}}} \frac{\partial q_{\mathrm{avg}}}{\partial T} \tag{S91}$$

$$= k_{\mathrm{B}} \left[\ln\left(\frac{q_{\mathrm{avg}}}{\theta} \right) + \langle \frac{E}{k_{\mathrm{B}} T} \rangle + 1 \right], \tag{S92}$$

where the thermal average energy is:

$$\langle \frac{E}{k_{\mathrm{B}} T} \rangle = \frac{1}{q_{\mathrm{avg}}} \frac{1}{M} \sum_{i} \sum_{\boldsymbol{k}} \frac{E_{i\boldsymbol{k}}}{k_{\mathrm{B}} T} \exp\left(-\frac{E_{i\boldsymbol{k}}}{k_{\mathrm{B}} T} \right). \tag{S93}$$

Finally, the differential entropy—which measures how the total entropy changes with the addition of one more particle at fixed temperature and volume—is calculated as:

$$S_{\mathrm{diff}} = \frac{\partial S}{\partial N} \tag{S94}$$

$$= \frac{\partial}{\partial N} \left\{ N k_{\mathrm{B}} \left[\ln\left(\frac{q_{\mathrm{avg}}}{\theta} \right) + \langle \frac{E}{k_{\mathrm{B}} T} \rangle + 1 \right] \right\} \tag{S95}$$

$$= k_{\mathrm{B}} \left[\ln\left(\frac{q_{\mathrm{avg}}}{\theta} \right) + \langle \frac{E}{k_{\mathrm{B}} T} \rangle \right]. \tag{S96}$$

This differential entropy is a particularly useful quantity for comparing with experimental adsorption data and other computational methods. It provides a quantum mechanical reference point that fully accounts for nuclear quantum effects in the hydrogen motion along the Pt(111) surface.

S4 Convergence Tests for Computational Parameters

To ensure numerical reliability of our results, we performed extensive convergence tests with respect to two critical computational parameters: k-point sampling density and the number of platinum layers in the slab model. These tests are essential for establishing the appropriate balance between computational accuracy and efficiency in our DFT and CNEO-DFT calculations. Because the additional quantum nuclear degrees of freedom within CNEO-DFT have very little impact on the convergence behavior of the electronic structure calculation with respect to calculation parameters and model setups, in the following the convergence tests are only reported for pure DFT calculations. Unshown data for CNEO-DFT indicate that CNEO-DFT has exactly the same behavior as conventional DFT for the convergence tests.

S4.1 Brillouin zone sampling

The proper sampling of the Brillouin zone is crucial for accurate electronic structure calculations in periodic systems. For hexagonal systems like the Pt(111) surface, Γ-centered k-point meshes are generally preferred to maintain proper symmetry in the reciprocal space. However, in the CP2K implementation, non-Γ-centered Monkhorst-Pack meshes can also be specified using an even number of k-points in each direction.

Figure S1 presents the convergence of our calculations with respect to k-point sampling, showing both absolute energies for hydrogen adsorption at the fcc and atop sites (Fig. S1(a)) and the energy difference between these sites (Fig. S1(b)). All tests were performed using a seven-layer Pt(111) slab model, with the bottom two layers fixed and the top five layers relaxed during the initial slab optimization with a $15 \times 15 \times 1$ k-point mesh. The optimized slabs were subsequently expanded to 3×3 supercells for hydrogen adsorption studies, with the platinum atoms fixed and only the hydrogen position optimized.

As evident from Figure S1, the $3 \times 3 \times 1$ k-point mesh is inadequate for accurately sampling

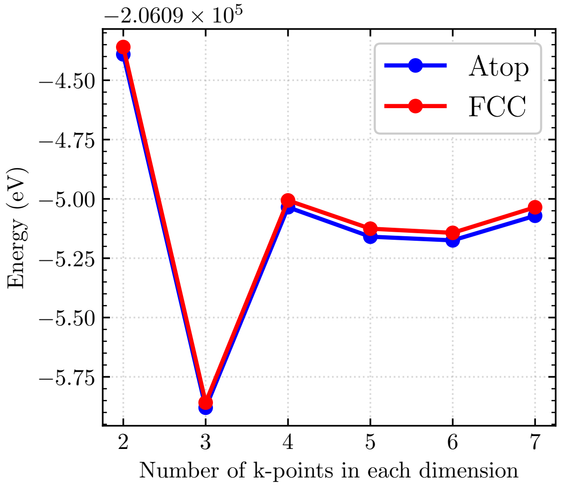

(a) Absolute energy

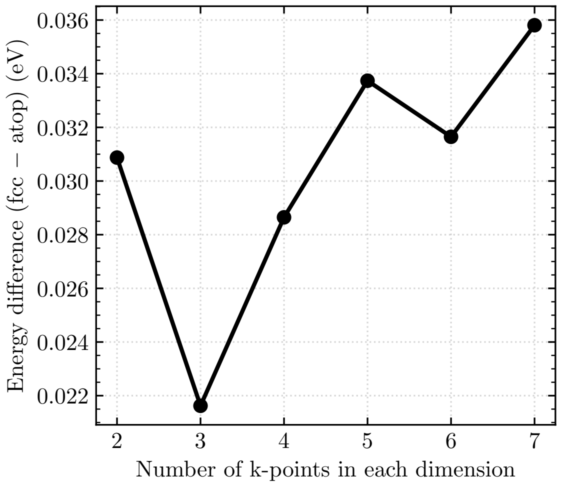

(b) Energy difference (fcc−atop)

Figure S1: Convergence with respect to k-point sampling for hydrogen adsorption on a seven-layer Pt(111) surface: (a) Absolute DFT energies for hydrogen adsorption at fcc and atop sites as a function of k-point mesh density; (b) Energy difference between fcc and atop adsorption sites. The $3 \times 3 \times 1$ k-point mesh is insufficient to adequately sample the Brillouin zone, while the $5 \times 5 \times 1$ mesh provides results nearly identical to the more computationally intensive $7 \times 7 \times 1$ mesh. For balance between accuracy and computational efficiency, the $5 \times 5 \times 1$ k-point mesh was selected for production calculations.

the 2D Brillouin zone of the 3×3 Pt(111) supercell. The results from the $5 \times 5 \times 1$ mesh, however, closely match those obtained with the denser $7 \times 7 \times 1$ mesh, with energy differences converged to within a few meV. This convergence behavior indicates that the $5 \times 5 \times 1$ k-point mesh offers an optimal balance between numerical accuracy and computational efficiency for the hydrogen adsorption systems studied in this work. All production calculations reported in the main text were therefore performed using this level of Brillouin zone sampling.

S4.2 Slab thickness

Another critical parameter for surface calculations is the thickness of the slab model, which must be sufficient to represent both the bulk-like behavior of the interior layers and the surface electronic structure correctly. Figure S2 shows the convergence of the energy difference between fcc and atop hydrogen adsorption sites as a function of the number of platinum

layers in the slab model.

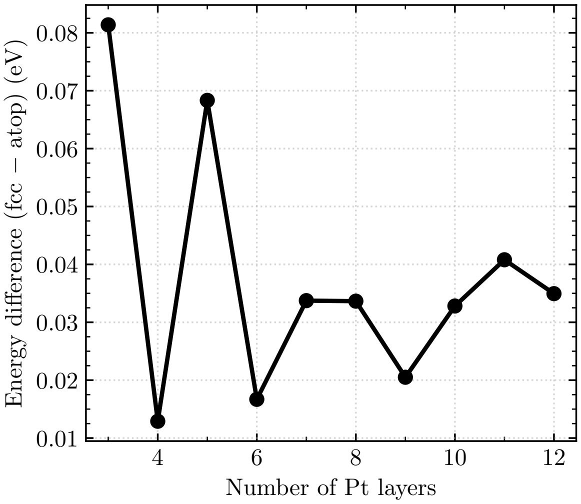

Figure S2: Convergence of the energy difference between fcc and atop hydrogen adsorption sites as a function of the number of platinum layers in the Pt(111) slab model. For each model, the bottom two layers were fixed at their bulk positions while the remaining layers were allowed to relax during the initial slab optimization with a 15 × 15 × 1 k-point mesh. The subsequent hydrogen adsorption calculations were performed on 3 × 3 supercells using a 5 × 5 × 1 k-point mesh with fixed platinum positions. The energy difference exhibits significant fluctuations for thinner slabs (three to five layers) but stabilizes with increasing thickness, with the seven-layer model showing converged behavior suitable for production calculations.

For each model tested, the bottom two layers were fixed at their bulk positions to mimic the underlying bulk crystal, while the remaining layers were allowed to relax during the initial slab optimization using a 15 × 15 × 1 k-point mesh. Following this optimization, the slabs were expanded to 3×3 supercells for hydrogen adsorption calculations using a $5 \times 5 \times 1$ k-point mesh, with the platinum atoms fixed and only the hydrogen position optimized.

As shown in Figure S2, the energy difference between adsorption sites exhibits pronounced fluctuations for thinner slabs (three to five layers), highlighting the importance of using sufficiently thick models for reliable surface calculations. With too few layers, the relative energetics of different adsorption sites can be severely affected, potentially leading to qualitatively incorrect conclusions about preferred adsorption configurations. The convergence behavior stabilizes with increasing slab thickness, with the seven-layer model demonstrating

consistent results that persist through further thickness increases (despite some fluctuations around 9 and 11 layers).

Based on these convergence tests, all production calculations reported in the main text employed the seven-layer Pt(111) slab model, which provides an optimal balance between computational cost and the accurate representation of surface electronic structure and adsorption energetics.

References

(S1) Marx, D.; Hutter, J. In *Modern Methods and Algorithms of Quantum Chemistry*, 2nd ed.; Grotendorst, J., Ed.; NIC Series; John von Neumann Institute for Computing: Jülich, 2000; Vol. 3; pp 329–477.

(S2) Lippert, G. R. Die GAPW-Dichtefunktional-Methode für Ab-Initio-Molekulardynamik-Simulationen. Dissertation, Universität Stuttgart, 1998.

(S3) Roetti, C. In *Quantum-Mechanical Ab-initio Calculation of the Properties of Crystalline Materials*; Pisani, C., Ed.; Springer Berlin Heidelberg: Berlin, Heidelberg, 1996; pp 125–137.

(S4) GitHub fork of PySCF with CNEO functionalities. `https://github.com/theorychemyang/pyscf`, Accessed May 23, 2025.

(S5) Lippert, G.; Hutter, J.; Parrinello, M. The Gaussian and Augmented-Plane-Wave Density Functional Method for Ab Initio Molecular Dynamics Simulations. *Theor. Chem. Acc.* **1999**, *103*, 124–140.

(S6) Krack, M.; Parrinello, M. All-Electron Ab-Initio Molecular Dynamics. *Phys. Chem. Chem. Phys.* **2000**, *2*, 2105–2112.
